# Ferrocenyl conjugated oxazepines/quinolines: multiyne coupling and ring–expanding or rearrangement

**DOI:** 10.3389/fchem.2024.1441539

**Published:** 2024-07-31

**Authors:** Yu Lei, Li Bao, Qiong Hu, Ke Zhang, Lingli Zong, Yimin Hu

**Affiliations:** College of Chemistry and Materials Science, Anhui Normal University, Wuhu, China

**Keywords:** hexadehydro-Diels-Alder reaction, ferrocene derivatives, benzyne, oxazepine, quinoline, rearrangement

## Abstract

Ferrocenyl conjugated oxazepine/quinoline derivatives were presented through the reaction of hexadehydro-Diels–Alder (HDDA) generated arynes with ferrocenyl oxazolines under mild conditions *via* ring-expanding or rearrangement processes. Water molecule participated in this unexpected rearrangement process to produce quinoline skeletons, and DFT calculations supported a ring-expanding and intramolecular hydrogen migration process for the formation of oxazepine derivatives. Two variants of this chemistry, expanded the reactivity between ferrocenyl conjugated substances and arynes, further providing an innovative approach for the synthesis of ferrocene derivatives.

## Introduction

**FIGURE 1:**
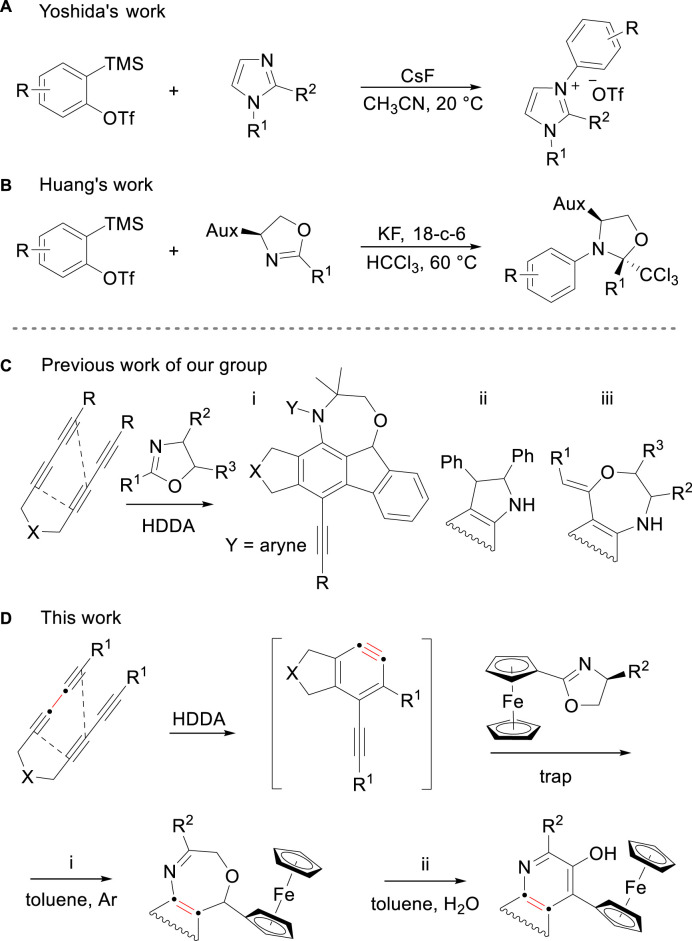
**(A, B)** Examples to produce *N*-arylation conjugates of benzynes and azaheterocycles. **(C)** Protocols for the synthesis of heterocycles from arynes and oxazolines. **(D)** This work, ferrocenyl conjugated oxazepines/quinolines.

Nitrogen is an efficient nucleophilic element in aryne chemistry (Arora et al., 2019; Pal et al., 2018). Therefore, azaheterocycles are often employed as neutral nucleophiles to trigger a diverse array of classical aryne-mediated coupling reactions to produce N-arylation conjugates (Yoshida et al., 2002; Huang et al., 2019; Zhou et al., 2018; Sun et al., 2017; Min et al., 2018; Bering et al., 2022). Yoshida and co-workers first demonstrated a protocol to generate *N*-alkyl-*N*′-arylimidazolium salts of *N*-substituted imidazoles with *o*-silylaryl triflates (Figure 1A). (Yoshida et al., 2002) Subsequently, Huang et al. investigated the reaction between oxazolines and arynes and observed the three-component coupling formation with chloroform to produce *N*-monoarylated products (Figure 1B). (Huang et al., 2019) It was not until 2020 that the unexpected transformation of the reaction by using aryne and oxazoline was revealed through a report by Zheng et al. compared to conventional N-arylation transformation (Figure 1C). (Zheng et al., 2020) Mechanistically, upon the cyclization of the initial substrate, the reaction between arynes and the C=N moiety of oxazolines produces benzazetidine ring which is described as a net [2 + 2] process by way of an initial, transient zwitterion (Yoshida et al., 2006; Arora et al., 2020) and then undergoes different reaction pathways to generate three variants of products due to the nature of the substituent at the two-position of the oxazoline. We envisioned that the protocol could be extended by using oxazoline bearing different substituents. A new compound containing iron and two cyclopentadienide ligands was reported in 1951 (Kealy and Pauson, 1951). Wilkinson and Fischer, later on, established its sandwich structure [Fe (*η* ^1^-C_5_H_5_)_2_] (Wilkinson et al., 1952). Ferrocene substituent has specific steric properties and well-defined and tunable redox behavior that makes it an attractive platform for biochemical and medicinal research (Fouda et al., 2007; Astruc, 2017; Patra and Gasser, 2017; Kowalski, 2018; Sharma and Kumar, 2021), and a variety of innovative achievements including electrochemistry, photoactive materials, and thermoelectrics have been sparkled over the past 7 decades (Ornelas et al., 2009; Garrigues et al., 2016; Pal et al., 2021; Walawalkar et al., 2021). Oxazepine and quinoline are valuable biologically skeletons among various pharmaceutical compounds (Michael, 2007; Karadeniz et al., 2021). In recent years, numerous studies have shown that ferrocenyl conjugated organic compound may exert enhanced or unexpected biological activities (Zora and Velioğlu, 2008; Kowalski, 2016). This inspired us to develop a protocol, ferrocenyl oxazolines were exploited to the system, reacted with arynes to afford ferrocenyl conjugated oxazepine/quinoline derivatives under different conditions (Figure 1D).

## Results and discussion

**SCHEME 1:**
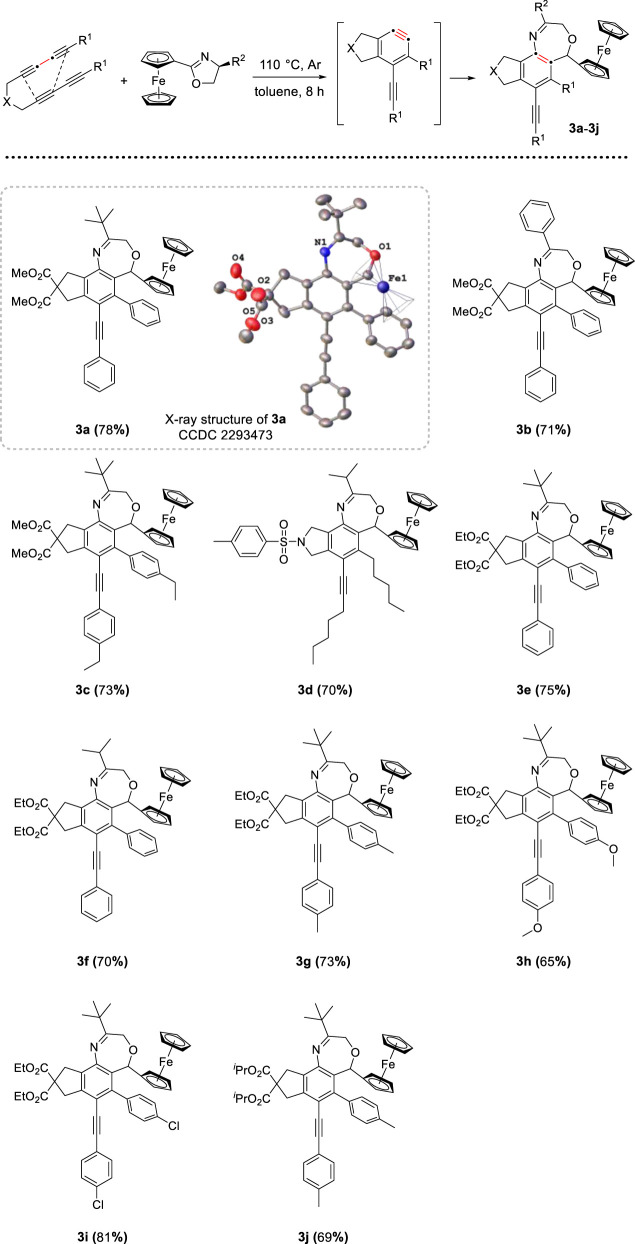
Preparation of oxazepine derivatives^[a,b]^. [a] Reaction conditions: tetraynes (1.0 mmol), oxazoline substrates (1.1 mmol), toluene (10 mL), stirred at 110°C under Ar atmosphere for 8 h. [b] Isolated yield.

As one class of the most highly reactive and versatile intermediates, arynes have found numerous applications in polycyclic aromatic functional framework (Karmakar and Lee, 2016; Fluegel and Hoye, 2021; Shi et al., 2021; Tan et al., 2024). Our study commenced with the investigation by using tetrayne precursor to generate a prototypical thermal aryne substrate (Hoye et al., 2012; Liu et al., 2019; Wang et al., 2021; Xu et al., 2023; Zhang et al., 2023), then reacted with (S)-(5-tert-butyloxazolidinyl) ferrocene. Under the argon atmosphere, ferrocenyl oxazoline and tetrayne were added to a Schlenk tube. The system was heated in a solution of toluene at 100°C overnight, after purification by column chromatography on neutral aluminum oxide, we obtained a seven-membered heterocycle compound 3a, the molecular structure was confirmed by X-ray diffraction (CCDC 2293473). After a brief screening of different reaction parameters, including molar ratio, temperature, and solvent, the optimal reaction conditions were determined as follows: 1.0 mmol tetrayne substrates and 1.1 equiv of ferrocenyl oxazolines were dissolved in 10 mL toluene under Ar atmosphere, heated at 110°C for 8 h. This protocol worked well and generated a series of target compounds smoothly without metal catalysts or other additives. To explore some of the generality of the reaction, we prepared a series of analogues of tetrayne substrates differing in substituents, and different substituents (*R* ^2^) of ferrocenyl oxazolines were also applied to this reaction. As shown in Scheme 1, a total of 10 functionalized benzoxazepine derivatives were obtained in moderate to good yield (ranging from 65% to 81%). Experimental results indicated that the yield of the synthesized product is higher in the presence of the electron-withdrawing group (e.g., *para*-Cl) on the benzene ring than the electron-donating group (e.g., *para*-Me, *para*-Et) (Yao et al., 2021; Lei et al., 2023), compound 3i was isolated with the highest yield (81%) among the examined substrates. Besides, oxazoline substrates bearing aromatic group (3b) or aliphatic group (3a and 3c) were reacted well, yielding the desired compounds.

**SCHEME 2:**
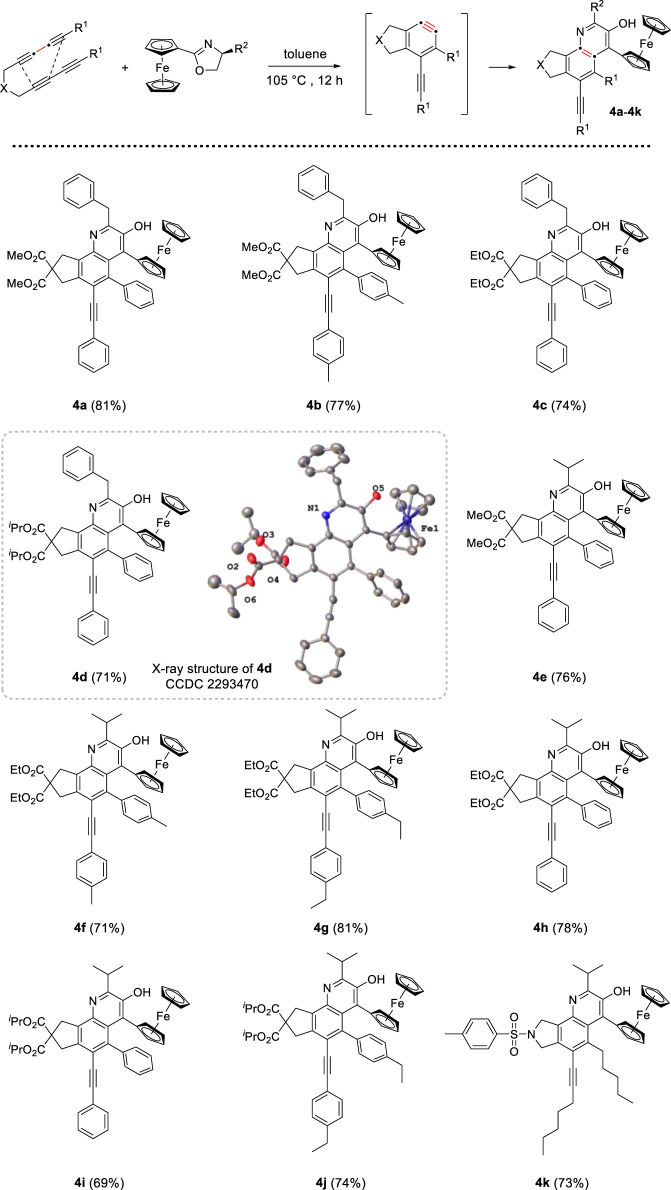
Preparation of quinoline derivatives^[a,b]^. [a] Reaction conditions: tetraynes (2.0 mmol), oxazoline substrates (1.0 mmol), H_2_O (1.0 mmol), toluene (10 mL), stirred at 105°C for 12 h. [b] Isolated yield.

Initially, the reaction system was proceeded under air atmosphere. The crude product seemed to decompose susceptibly in silica gel and oxazepine derivative was not obtained, but a small amount of quinoline derivatives were isolated. Based on the molecular structure of product, we speculated that water molecule participated in the unexpected rearrangement process to construct quinoline skeleton. After screening, the following standard conditions were identified, these reactions were performed with 1.0 mmol ferrocenyl oxazolines, 2.0 equiv of tetrayne substrates and 1.0 equiv of H_2_O, dissolved all reactants in toluene and heated at 105°C for 12 h. In addition, the cascade process was insensitive to air. With the optimized reaction conditions in hand, we investigated the scope of two reactants for the generation of quinoline derivatives. As depicted in Scheme 2, different tetraynes were subjected to this transformation, the corresponding products were obtained smoothly in moderate to good yields (69%–81%). Ferrocenyl oxazolines containing benzyl and isopropyl were suitable for this protocol. The tether (X) in tetrayne was expanded to nitrogen, and target compound 4k was generated successfully with good yield. Furthermore, we confirmed the molecular structure of compound 4d by X-ray diffraction analysis (CCDC 2293470).

**SCHEME 3:**
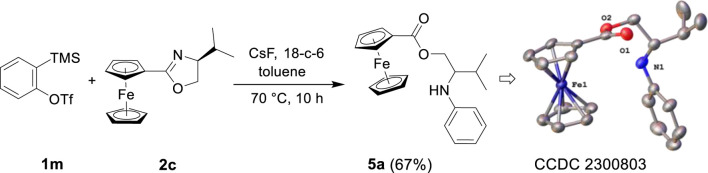
Reaction of Kobayashi-derived benzyne and oxazoline^[a,b]^.[a]Reaction conditions: 1m (1.0 mmol), 2c (1.0 mmol), 18-crown-6 (2.0 mmol), CsF (2.0 mmol), toluene (5 mL), stirred at 70°C for 10 h. [b] Isolated yield.

Sometimes, different method-derived arynes were captured by the same reagent, but completely different products were generated (Zhang et al., 2016; Yao et al., 2020). Therefore, we attempted to apply Kobayashi-derived arynes in this reaction (Himeshima et al., 1983). Through screening, the most suitable reaction conditions were determined as follows: mixed oxazoline 2c (1.0 mmol), benzyne precursor 1m (1.0 equiv), 18-crown-6 (2.0 equiv), and CsF (2.0 equiv) as the fluoride source in toluene, reacted at 70°C for 10 h. As shown in Scheme 3, 1m was induced by fluoride to remove TMS, while the detachment of its adjacent OTf group to produce benzyne intermediate, and then trapped by 2c that is present *in situ*. Likely because the pristine environment for the generation of benzyne intermediate results in different reactivities to produce ester derivative. By combining the X-ray diffraction structure of compound 5a (CCDC 2300803), we speculated that water molecule was involved in this reaction. The ability to access this class of reactive aryne intermediate in different environments has led to a new type of trapping reaction compared with previous experiments. Further work on the expansion and mechanism of this protocol is in progress.

**FIGURE 2:**
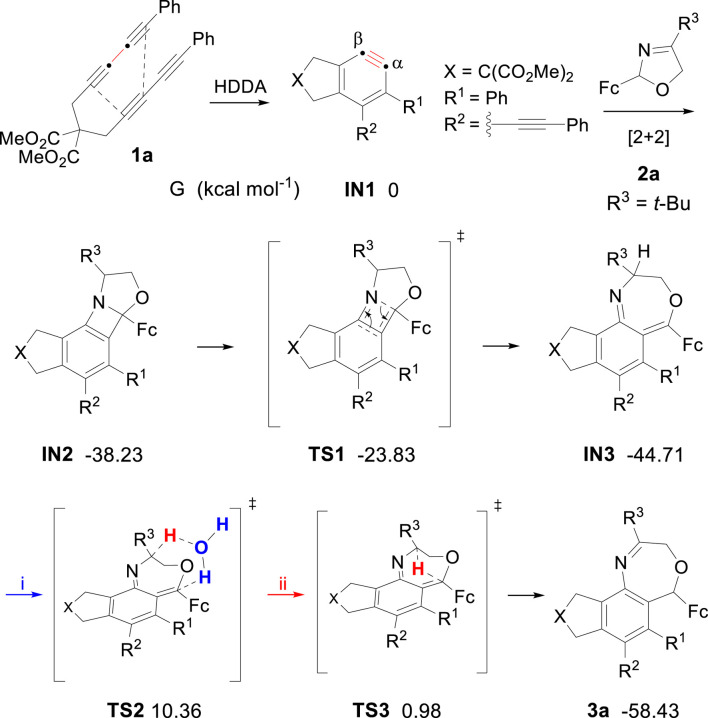
Relative free-energy profiles for generating oxazepine derivatives.

We performed density functional theory (DFT) calculations to reveal the mechanistic details for generating oxazepine derivatives (Figure 2). The lowest energy conformer for aryne intermediate IN1 is set to G = 0 kcal mol^−1^. In principle, reactions of a net [2 + 2] process *via* C≡C bond and C=N bond, once formed, are generally in a nonselective fashion. However, we were surprised to observe that only one major product was generated. A reasonable explanation is that the *β*-carbon on the alkyne bond is preferentially attacked by nitrogen atom due to electric effect (Karmakar et al., 2014). Then the benzazetidine skeleton IN2 was converted by ring opening into its valence tautomer IN3 through TS1. In general, hydrogen migration process might occur through two pathways, and the free energy were computed to be (i) 10.36 kcal mol^-1^ through water-assisted intermolecular hydrogen migration and (ii) 0.98 kcal mol^-1^ through intramolecular hydrogen migration, individually. Thus, based on the reaction condition of experiment, intramolecular hydrogen migration mechanism is offered as the most reasonable rationale to account for the generation of compound 3a. Notably, the energy barrier that IN3 needs to overcome when producing products through TS3 is 45.69 kcal mol^-1^.

**FIGURE 3:**
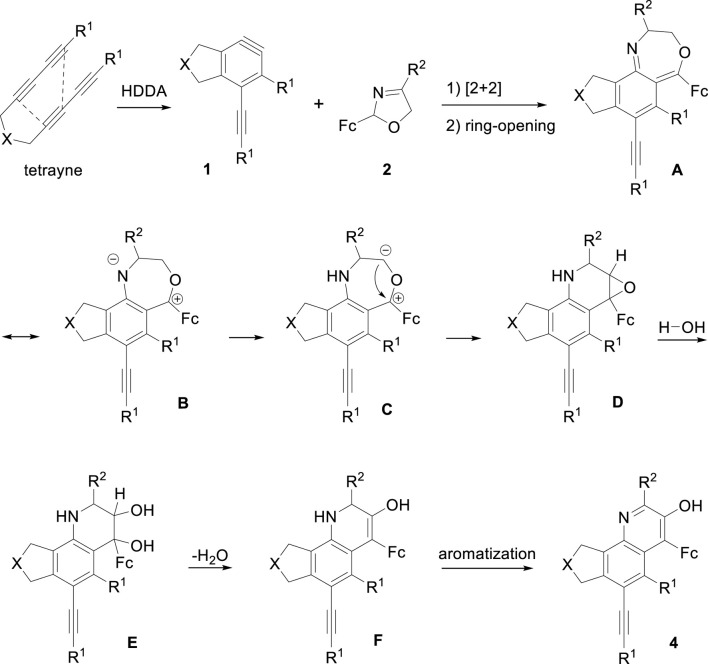
Proposed mechanism for generating quinoline derivatives.

Next, we conducted a control experiment to gain mechanistic insights into the formation of quinoline derivatives. Compound 3a was dissolved in toluene in the presence of H_2_O and heated at 105°C overnight, the transformation did not occur. Presumably because 3a was relatively stable, which was distinct from 1,4-oxazepine possessing anti-aromatic character (Kurita et al., 1987; Cheng et al., 2015; Cheng et al., 2017). Based on the experimental result, we proposed a plausible rearrangement process of this reaction. As shown in Figure 3, the initial reaction processes were consistent with the generation of oxazepine, tetrayne substrate engaged in thermodynamic cycloisomerization to produce aryne intermediate 1, which then generated with oxazoline 2 to form intermediate A, accompanied by resonance B *via* electron delocalization. Due to the instability of negatively charged nitrogen atom, deprotonation‒protonation occurred to form C. Then carbanion combined with carbocation to form epoxide intermediate D, which underwent ring-opening reaction and trapped with H_2_O to give E. Next, the dehydration of E led to the intermediate F, followed by the aromatization process, generating quinoline derivatives 4.

**FIGURE 4:**
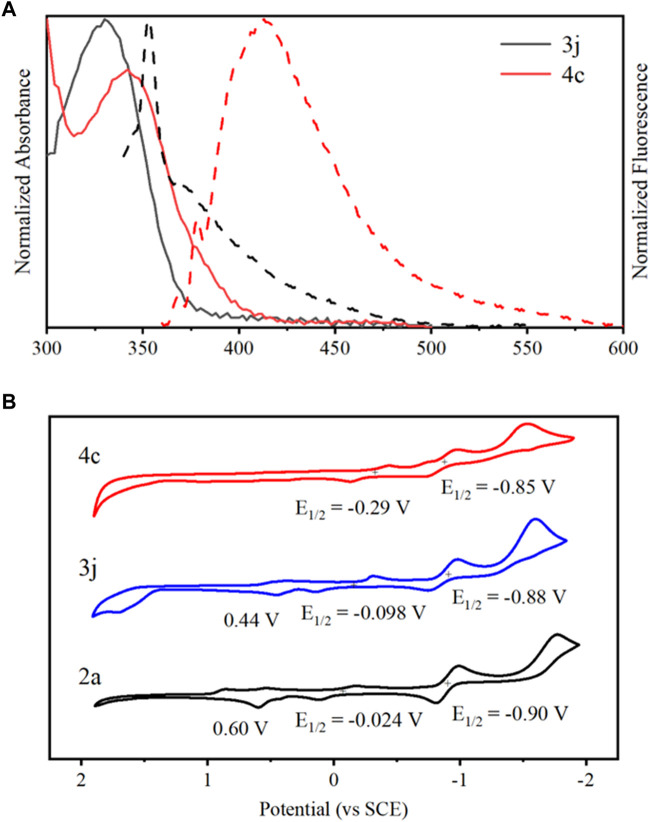
**(A)** Overlaid normalized absorption spectra (solid line) and fluorescence spectra (broken line) of 3j and 4c in acetonitrile. **(B)** Cyclic voltammograms of 2a (black), 3j (blue), and 4c (red) in acetonitrile in the presence of 0.1 M TBAPF_6_ at rt.

The photophysical properties of 3j and 4c were investigated in three different solvents, experimental results indicated that these compounds were insensitive to solvent polarity, and the data were summarized in Supplementary Table S1. The maximum absorption peak of each compound is 330 and 342 nm in acetonitrile, whereas gives emission maxima at 353 and 413 nm, respectively (Figure 4A). In contrast with 3j, 4c showed a slight red-shift in absorption and emission spectra, presumably due to the increasing conjugation of planar quinoline ring, resulting in conformation restriction (Wang et al., 2015; Wang et al., 2023). Next, the electrochemical behavior of the representative compounds was studied by cyclic voltammetry at a glassy carbon electrode in acetonitrile containing TBAPF_6_ as the supporting electrolyte (Figure 4B), while the corresponding electrochemical data are presented in Supplementary Table S2. Substance 2a displayed an irreversible oxidation with potentials at 0.60 V, as well as two reversible reductions, at −0.024 V and −0.90 V, respectively. Comparatively, 3j and 4c showed the similar reversible reduction potentials, but with slight changes. The first oxidation potentials of 3j slightly decreased, but it was not observed in 4c.

## Conclusion

In summary, we have developed an efficient and facile synthetic method for accessing oxazepine/quinoline skeletons through the reaction of aryne and ferrocenyl oxazoline. These protocols exhibited several unique characteristics, including a broad substrate scope, mild reaction condition, and unexpected ring-contracting rearrangement. DFT calculations supported an intramolecular hydrogen migration process for the formation of oxazepine derivatives. Furthermore, the photophysical and electrochemical properties of representative compounds were studied. Experimental results have also revealed that the reactivity of oxazolines bearing ferrocene group was different from those of other substituted oxazolines. Our future studies will be focused on expanding the scope of these protocols as well as the reactivity of ferrocenyl conjugated substances in organic synthesis.

## Methods

### Procedure for oxazepine derivatives

Tetraynes (1.0 mmol) and ferrocenyl oxazolines (1.1 mmol) were mixed in an oven-dried Schlenk tube (50 mL) equipped with a magnetic stir bar and heated in a 110°C oil bath in 10 mL toluene for 8 h under argon atmosphere. Then the reaction mixture was cooled to room temperature, quenched with saturated NaCl, and extracted with ethyl acetate (3 × 10 mL). The combined organic extracts were dried over anhydrous MgSO_4_, filtered, and concentrated under reduced pressure. The crude product was purified by column chromatography on neutral aluminum oxide (petroleum ether: EtOAc = 40:1) to yield compounds 3a-3j.

### Procedure for quinoline derivatives

Tetraynes (2.0 mmol), ferrocenyl oxazolines (1.0 mmol) and H_2_O (1.0 mmol) were mixed in an oven-dried Schlenk tube (50 mL) equipped with a magnetic stir bar and heated in a 105°C oil bath in 10 mL toluene for 12 h under air atmosphere. Then the reaction mixture was cooled to room temperature, quenched with saturated NaCl, and extracted with ethyl acetate (3 × 10 mL). The combined organic extracts were dried over anhydrous MgSO_4_, filtered, and concentrated under reduced pressure. The crude product was purified by column chromatography on silica gel (petroleum ether: EtOAc = 60:1) to yield compounds 4a-4k.

## Data Availability

The original contributions presented in the study are included in the article/Supplementary Material, further inquiries can be directed to the corresponding author.
