# Supplementary material for: Ferrocenyl conjugated oxazepines/quinolines: multiyne coupling and ring–expanding or rearrangement
Source: Front Chem. 2024 Jul 31;12:1441539. doi: 10.3389/fchem.2024.1441539 (PMC11322062; doi:10.3389/fchem.2024.1441539)
Supplement: Supplementary file 1 [file DataSheet1.docx]

**Supplementary Information**

**Ferrocenyl conjugated** **oxazepines/quinolines: multiyne coupling and ring-expanding or rearrangement**

Yu Lei, Li Bao, Qiong Hu, Ke Zhang, Lingli Zong and Yimin Hu*

*College of Chemistry and Materials Science, Anhui Normal University,*

*Wuhu, Anhui 241002, China E-mail: yiminhu@ahnu.edu.cn*

**Contents**

1. General Experimental Procedures.………….………………………………….……………S2

2. Characterization Data for the New Compounds……………………….……………………S5

3. X-Ray Structure for **3a,** **4d**, and **5a**..……………………………………………….………S23

4. Computational Details.……………………….……………………………………………S26

5. Photophysical data.……………………….……………………………………………S53

6. Electrochemical data.……………………….……………………………………………S54

7. ^1^H and ^13^C Spectra for New Compounds.……………………………………….…………S55

**1. General experimental procedures**

All the catalytic reactions were performed under an argon atmosphere using the oven-dried Schlenk flask. The chemicals were purchased from Adamas-beta, Macklin reagent, and Bidepharm. All solvents and materials were pre-dried, redistilled or recrystallized before use. ^1^H NMR (400 MHz), ^13^C NMR (101 MHz) and spectra were recorded on a Bruker Avance 400 spectrometer with CDCl_3_ or C_6_D_6_ as the solvent. ^1^H NMR (500 MHz) and ^13^C NMR (125 MHz) spectra were recorded on a Bruker Avance 500 spectrometer with C_6_D_6_ as the solvent. Chemical shifts are reported in ppm by assigning TMS resonance in the ^1^H NMR spectra as 0.00 ppm and CDCl_3_ resonance in the ^13^C spectra as 77.0 ppm and C_6_D_6_ resonance in the ^13^C spectra as 128.0 ppm. Data for ^1^H NMR are reported as follows: chemical shift (δ ppm), multiplicity (s = singlet, d = doublet, t = triplet, q = quartet, m = multiplet), coupling constant (Hz) and integration. Data for ^13^C NMR are recorded with broad-band proton decoupling technique and are reported in terms of chemical shift. Column chromatography was performed on silica gel 300–400 mesh/ aluminum oxide, neutral 200-300 mesh. Thin-layer chromatography (TLC) was performed on silica gel plates (HSGF 254). Melting points were determined using a Gallenkamp melting point apparatus and are uncorrected. The FT-IR spectra were recorded from KBr pellets or thin film from CHCl_3_ on the NaCl window in the 4000-400 cm^-1^ ranges on a Nicolet 5DX spectrometer. High-resolution mass spectra were recorded on Shimadzu LCMS-9030.

**X-ray structure analysis**. Crystal of compounds **3a**, **4d** and **5a** suitable for X-ray analysis were obtained via the slow diffusion of petroleum ether into their ethyl acetate solution. The vial containing this solution was placed, loosely capped, to promote the crystallization. A suitable crystal was chosen and mounted on a glass fiber using grease. Data were collected using a Bruker APEX-II CCD diffractometer operating at T = 293(2) K. The determination of unit cell parameters and data collections were performed with Mo Kα radiation (λ) at 0.71073 Å. The total number of runs and images was based on the strategy calculation from the program APEX2 (Bruker). The structure was solved by the structure solution program Olex2 (Dolomanov et al., 2009), and the model was refined with version 2014/7 of ShelXL (Sheldrick, 2015) using full matrix least squares on F^2^ minimization.

**Absorption and emission measurements**. UV-visible absorption and fluorescence emission spectra were recorded on commercial spectrophotometers (Shimadzu UV-2450 and Edinburgh FS5 spectrometers, 190-900 nm scan range) at room temperature (10 mm quartz cuvette). Relative fluorescence quantum efficiencies of **3j** and **4c** were obtained by comparing the areas under the corrected emission spectrum of the test sample in various solvents with quinine sulfate in 0.05 M H_2_SO_4_ aqueous solution (Φ = 0.54).

**Electrochemical measurements**. Cyclic voltammograms of 1.0 mM **2a**, **3j**, and **4c** were measured in acetonitrile solution, containing 0.1 M TBAPF_6_ as the supporting electrolyte, glassy carbon electrode as a working electrode, Pt wire as a counter electrode, and saturated calomel electrode (SCE) as reference electrode at 100 mV s ^-1^ of scanning rate at room temperature.

**General procedure for preparation of tetraynes** **^[1-3]^
Preparation of catalyst Pd(PPh_3_)_2_Cl_2_**

3.54 g PdCl_2_ and 4 g LiCl were mixed in a 500 mL three-necked flask with 150-200 mL methanol as solvent, magnetically stirred and heated in oil bath at 50-60 °C. After the solid was dissolved, 25 mL of THF (removed water with sodium wire) containing 13.1 g PPh_3_ were added in the above three-necked flask, and the color of the solution changed from brown to yellow, reflux reaction for 3 h. After reaction solution cooled, filtered and washed with anhydrous ethanol, yellow solid Pd(PPh_3_)_2_Cl_2_ catalyst was obtained finally.

**Preparation of diyne substrates**

10 g NaH (60%) and 200-300 mL acetonitrile were added in 500 mL three-necked flask with magnetic stirring. 100 mmol malonate and 30 g 3-bromopropyne (98%) were added in the above 500 mL three-necked flask dropwise in turn by separatory funnel, magnetically stirred for 8-10 h under ice-water bath. The organic phase was extracted with ethyl acetate and dried with anhydrous MgSO_4_. The solvent was evaporated in vacuo and diyne substrates as white solid were obtained finally.

**Preparation of brominated alkynes**

10.68 g 1-bromopyrrolidine-2,5-dione (NBS), 0.43 g AgNO_3_, and 50 mmol phenylacetylene or substituted phenylacetylene or alkyl alkyne were added in 250 mL three-necked flask in turn, 100 mL acetone as a solvent, magnetically stirred at room temperature for 3 h. The organic phase was extracted with n-hexane and dried with anhydrous MgSO_4_. The solvent was evaporated in vacuo and brominated alkynes compound as brown solid were obtained finally.

**Preparation of tetrayne substrates**

0.3 g Pd(PPh_3_)_2_Cl_2_, 0.25 g CuI and 20 mmol diyne substrate were added in 500 mL three-necked flask, protected with anhydrous anaerobic conditions under argon. After 0.5 h, 200-300 mL acetonitrile, 8.08 g Et_3_N and 50 mmol brominated aryl alkyne were added in turn, magnetically stirred for 10-12 h under ice-water bath. The organic phase was extracted with ethyl acetate and dried with anhydrous MgSO_4_. It was separated by column chromatography on silica gel to obtain tetrayne substrate as yellow solid finally.

**Preparation of 4-methyl-N-(prop-2-yn-1-yl) benzenesulfonamide**

50 mmol 4-methylbenzene-1-sulfonyl chloride, 50 mmol prop-2-yn-1-amine and 200 mL CH_2_Cl_2_ were added in 500 mL three-necked flask. After 0.5 h, 50 mmol Et_3_N were added, magnetically stirred under ice-water bath for 4 h. The solvent was evaporated in vacuo to obtain the product as yellow solid finally.

**Preparation of 4-methyl-N, N-di(prop-2-yn-1-yl) benzenesulfonamide**

50 mmol 4-methyl-N-(prop-2-yn-1-yl) benzenesulfonamide, 55 mmol PPh_3_, 200 mL tetrahydrofuran (THF) as solvent were added in 500 mL three-necked flask, protected with anhydrous anaerobic conditions under argon. After 0.5 h, 55 mmol prop-2-yn-1-ol and 50 mmol diethyl azodicarboxylate (DEAD) were added dropwise by constant pressure funnel in turn, magnetically stirred under ice-water bath, reaction for 12 h. The organic phase was extracted with ethyl acetate and dried with anhydrous MgSO_4_. It was separated by column chromatography on silica gel to obtain the product as yellow solid finally.

**Preparation of N, N-di(deca-2,4-diyn-1-yl)-4-methylbenzenesulfonamide**

0.35 g CuCl, 0.14 g NH_2_OH•HCl and 10 mmol 4-methyl-*N*, *N*-di(prop-2-yn-1-yl) benzenesulfonamide were added in 500 mL three-necked flask, protected with anhydrous anaerobic conditions under argon. After 0.5 h, 45 mL 30% *n*-BuNH_2_ aqueous solution (13.5 g *n*-BuNH_2_ + 32 mL H_2_O) and 30 mmol 1-bromohept-1-yne (freshly prepared) was added dropwise by the constant pressure funnel in turn, 100-150 mL CH_2_Cl_2_ as solvent, magnetically stirred under ice-water bath, reaction for 10-12 h. The organic phase was extracted with ethyl acetate and dried with anhydrous MgSO_4_. It was separated by column chromatography on silica gel to obtain the product as yellow oil finally.

**References**

1. Hoye, T. R., Baire, B., Niu, D., Willoughby, P. K., and Woods, B. P. (2012). The hexadehydro-Diels–Alder reaction. *Nature* 490, 208‒212.
2. Zheng, X., Liu, B., Yang, F., Hu, Q., Yao, L., and Hu, Y. (2020). Access to benzoxazepines and fully substituted indoles via HDDA coupling. *Org. Lett.* 22, 956–959.
3. Yao, L., Hu, Q., Bao, L., Zhu, W., and Hu, Y. (2021). Fully substituted conjugate benzofuran core: multiyne cascade coupling and oxidation of cyclopropenone. *Org. Lett.* 23, 4971−4975.

**2. Preparation and characterization data for all compounds**

**Preparation of compound 3a**

Tetrayne **1a** (1.0 mmol), compound **2a** (1.1 mmol), were mixed in an oven-dried Schlenk tube (50 mL) equipped with a magnetic stir bar and heated in a 110 ºC oil bath in 10 mL toluene for 8 hours under argon atmosphere. Then the reaction mixture was cooled to room temperature, quenched with saturated NaCl, and extracted with ethyl acetate (3 × 10 mL). The combined organic extracts were dried over anhydrous MgSO_4_, filtered, and concentrated under reduced pressure. The crude product was purified by column chromatography on neutral aluminum oxide (petroleum ether : EtOAc = 40:1) to yield the product **3a** (560 mg, 78%) as an orange solid.

**Data for 3a**

**^1^H NMR** (500 MHz, C_6_D_6_): δ 7.49 (d, *J* = 8.1 Hz, 1H), 7.37 (d, *J* = 7.4 Hz, 1H), 7.01-6.97 (m, 2H), 6.94-6.92 (m, 2H), 6.89-6.88 (m, 1H), 6.66-6.63 (m, 3H), 6.10 (s, 1H), 4.22 (d, *J* = 17.5 Hz, 1H), 4.19-4.18 (m, 1H), 4.03 (d, *J* = 17.5 Hz, 1H), 3.92 (d, *J* = 17.5 Hz, 1H), 3.79 (d, *J* = 12.0 Hz, 1H), 3.65 (d, *J* = 17.5 Hz, 1H), 3.59-3.58 (m, 1H), 3.44 (d, *J* = 12.1 Hz, 2H), 3.41 (s, 1H), 3.30 (s, 5H), 2.98 (s, 3H), 2.93 (s, 3H), 0.72 (s, 9H).

**^13^C NMR** (101 MHz, C_6_D_6_): δ 178.4, 170.7, 170.7, 145.3, 143.3, 142.6, 138.2, 133.9, 130.6, 130.3, 129.0, 127.3, 127.1, 122.6, 115.7, 96.0, 88.5, 87.0, 73.8, 68.5, 67.7, 67.0, 66.3, 65.3, 58.6, 58.1, 51.2, 40.8, 38.7, 38.3, 26.2.

**IR** (neat): 2953, 1737, 1597, 1365, 1271, 1252, 1069, 760, 706, 692 cm^-1^.

**HRMS**: m/z calcd for C_44_H_41_FeNO_5_ [M+H]^+^ : 720.2407, found: 720.2411.

**mp:** 216‒218 ºC.

**Preparation of compound 3b**

Tetrayne **1a** (1.0 mmol), compound **2b** (1.1 mmol), were mixed in an oven-dried Schlenk tube (50 mL) equipped with a magnetic stir bar and heated in a 110 ºC oil bath in 10 mL toluene for 8 hours under argon atmosphere. Then the reaction mixture was cooled to room temperature, quenched with saturated NaCl, and extracted with ethyl acetate (3 × 10 mL). The combined organic extracts were dried over anhydrous MgSO_4_, filtered, and concentrated under reduced pressure. The crude product was purified by column chromatography on neutral aluminum oxide (petroleum ether : EtOAc = 40:1) to yield the product **3b** (524 mg, 71%) as an orange solid.

**Data for 3b**

**^1^H NMR** (500 MHz, C_6_D_6_): δ 7.83 (d, *J* = 6.2 Hz, 2H), 7.64 (dd, *J* = 11.8, 7.8 Hz, 2H), 7.31-7.25 (m, 3H), 7.19 (s, 2H), 7.11 (d, *J* = 6.6 Hz, 3H), 6.92 (s, 3H), 6.44 (s, 1H), 4.59 (d, *J* = 12.1 Hz, 1H), 4.45 (d, *J* = 17.5 Hz, 1H), 4.40 (s, 1H), 4.35 (d, *J* = 17.5 Hz, 1H), 4.23 (d, *J* = 17.5 Hz, 1H), 4.16 (d, *J* = 12.1 Hz, 1H), 4.02 (d, *J* = 17.5 Hz, 1H), 3.65 (s, 5H), 3.64-3.60 (m, 3H), 3.27 (s, 3H), 3.26 (s, 3H).

**^13^C NMR** (101 MHz, C_6_D_6_): δ 172.1, 172.0, 169.0, 145.9, 144.7, 144.0, 139.9, 138.1, 136.2, 131.8, 131.5, 130.9, 130.3, 130.1, 128.5, 123.9, 117.7, 97.9, 90.9, 88.3, 75.6, 69.2, 68.7, 67.3, 67.0, 67.0, 61.7, 59.6, 52.6, 42.0, 40.2.

IR (neat): 2952, 1738, 1569, 1435, 1280, 1251, 1103, 758, 691 cm^-1^.

**HRMS**: m/z calcd for C_46_H_37_FeNO_5_ [M+H]^+^ : 740.2094, found: 740.2096.

**mp**: 205‒207 ºC.

**Preparation of compound 3c**

Tetrayne **1b** (1.0 mmol), compound **2a** (1.1 mmol), were mixed in an oven-dried Schlenk tube (50 mL) equipped with a magnetic stir bar and heated in a 110 ºC oil bath in 10 mL toluene for 8 hours under argon atmosphere. Then the reaction mixture was cooled to room temperature, quenched with saturated NaCl, and extracted with ethyl acetate (3 × 10 mL). The combined organic extracts were dried over anhydrous MgSO_4_, filtered, and concentrated under reduced pressure. The crude product was purified by column chromatography on neutral aluminum oxide (petroleum ether : EtOAc = 40:1) to yield the product **3c** (565 mg, 73%) as an orange solid.

**Data for 3c**

**^1^H NMR** (400 MHz, C_6_D_6_): δ 7.79 (dd, *J* = 7.8, 1.8 Hz, 1H), 7.66 (dd, *J* = 7.7, 1.8 Hz, 1H), 7.26 (s, 1H), 7.24 (s, 1H), 7.22-7.17 (m, 2H), 6.84 (d, *J* = 8.2 Hz, 2H), 6.48 (s, 1H), 4.56 (d, *J* = 17.5 Hz, 1H), 4.50-4.48 (m, 1H), 4.34 (d, *J* = 17.5 Hz, 1H), 4.24 (d, *J* = 17.5 Hz, 1H), 4.10 (d, *J* = 11.9 Hz, 1H), 3.96 (d, *J* = 17.5 Hz, 1H), 3.89-3.88 (m, 1H), 3.78-3,73 (m, 3H), 3.62 (s, 5H), 3.26 (s, 3H), 3.22 (s, 3H), 2.49 (q, *J* = 7.6 Hz, 2H), 2.30 (q, *J* = 7.6 Hz, 2H), 1.12 (t, *J* = 7.6 Hz, 3H), 1.02 (s, 9H), 0.97 (t, *J* = 7.6 Hz, 3H).

**^13^C NMR** (101 MHz, C_6_D_6_): δ 179.6, 172.2, 172.1, 146.6, 144.7, 144.5, 143.9, 143.8, 137.1, 135.1, 132.2, 131.9, 130.4, 128.8, 127.6, 121.4, 117.5, 97.6, 90.1, 88.1, 75.2, 69.9, 69.1, 68.5, 67.7, 66.7, 60.0, 59.6, 52.6, 52.6, 42.2, 40.1, 39.7, 29.0, 27.6, 16.1, 15.5.

**IR** (neat): 2965, 1736, 1596, 1365, 1274, 1250, 1071, 838, 777 cm^-1^.

**HRMS**: m/z calcd for C_48_H_49_FeNO_5_ [M+H]^+^ : 776.3033, found: 776.3041.

**mp**: 173‒174 ºC.

**Preparation of compound 3d**

Tetrayne **1c** (1.0 mmol), compound **2c** (1.1 mmol), were mixed in an oven-dried Schlenk tube (50 mL) equipped with a magnetic stir bar and heated in a 110 ºC oil bath in 10 mL toluene for 8 hours under argon atmosphere. Then the reaction mixture was cooled to room temperature, quenched with saturated NaCl, and extracted with ethyl acetate (3 × 10 mL). The combined organic extracts were dried over anhydrous MgSO_4_, filtered, and concentrated under reduced pressure. The crude product was purified by column chromatography on neutral aluminum oxide (petroleum ether : EtOAc = 40:1) to yield the product **3d** (512 mg, 70%) as an orange solid.

**Data for 3d**

**^1^H NMR** (500 MHz, C_6_D_6_): δ 7.79 (d, *J* = 8.2 Hz, 2H), 6.70 (d, *J* = 8.1 Hz, 2H), 6.37 (s, 1H), 4.94 (s, 2H), 4.86-4.83 (m, 1H), 4.79-4.76 (m, 1H), 4.61 (q, *J* = 13.2 Hz, 2H), 4.42 (dd, *J* = 11.4, 6.1 Hz, 1H), 4.09 (dd, *J* = 11.4, 5.0 Hz, 1H), 4.02 (t, *J* = 1.9 Hz, 2H), 3.96 (s, 5H), 3.41 (s, 1H), 2.93-2.85 (m, 2H), 2.18 (t, *J* = 6.7 Hz, 2H), 1.83 (s, 3H), 1.69-1.63 (m, 2H), 1.52-1.47 (m, 2H), 1.43-1.25 (m, 8H), 0.97 (t, *J* = 7.3 Hz, 3H), 0.89 (d, *J* = 3.1 Hz, 3H), 0.87 (d, *J* = 3.0 Hz, 3H), 0.82 (t, *J* = 7.1 Hz, 3H).

**^13^C NMR** (125 MHz, C_6_D_6_): δ 171.6, 145.5, 142.8, 135.3, 135.0, 129.8, 128.4, 127.7, 119.7, 104.6, 97.6, 77.9, 71.7, 71.6, 70.6, 70.5, 70.0, 64.1, 57.5, 55.1, 54.6, 31.2, 31.1, 30.0, 29.5, 23.5, 22.2, 21.1, 19.5, 19.2, 18.3, 14.3, 13.7.

**IR** (neat): 2961, 2231, 1735, 1587, 1343, 1282, 1241, 1054, 848, 761 cm^-1^.

**HRMS**: m/z calcd for C_43_H_52_FeN_2_O_3_S [M+H]^+^ : 733.3121, found: 733.3128.

**mp**: 227‒229 ºC.

**Preparation of compound 3e**

Tetrayne **1d** (1.0 mmol), compound **2a** (1.1 mmol), were mixed in an oven-dried Schlenk tube (50 mL) equipped with a magnetic stir bar and heated in a 110 ºC oil bath in 10 mL toluene for 8 hours under argon atmosphere. Then the reaction mixture was cooled to room temperature, quenched with saturated NaCl, and extracted with ethyl acetate (3 × 10 mL). The combined organic extracts were dried over anhydrous MgSO_4_, filtered, and concentrated under reduced pressure. The crude product was purified by column chromatography on neutral aluminum oxide (petroleum ether : EtOAc = 40:1) to yield the product **3e** (560 mg, 75%) as an orange solid.

**Data for 3e**

**^1^H NMR** (500 MHz, C_6_D_6_): δ 7.80 – 7.78 (m, 1H), 7.66 (d, *J* = 7.3 Hz, 1H), 7.28 (q, *J* = 6.9 Hz, 2H), 7.23-7.21 (m, 2H), 7.19 (s, 1H), 6.96-6.90 (m, 3H), 6.39 (s, 1H), 4.56 (d, *J* = 17.4 Hz, 1H), 4.48 (s, 1H), 4.34 (d, *J* = 17.5 Hz, 1H), 4.26 (d, *J* = 17.4 Hz, 1H), 4.08 (d, *J* = 12.0 Hz, 1H), 4.01 (d, *J* = 17.4 Hz, 1H), 3.98-3.95 (m, 1H), 3.94-3.93 (m, 1H), 3.92-3.90 (m, 1H), 3.88 (d, *J* = 2.0 Hz, 1H), 3.87-3.82 (m, 1H), 3.74-3.70 (m, 3H), 3.60 (s, 5H), 1.01 (s, 9H), 0.87 (t, *J* = 6.9 Hz, 3H), 0.86 (t, *J* = 7.3 Hz, 3H).

**^13^C NMR** (125 MHz, C_6_D_6_): δ 179.7, 171.8, 171.7, 146.8, 144.6, 144.2, 139.7, 135.4, 132.1, 131.7, 130.4, 128.6, 128.5, 128.3, 124.1, 117.1, 97.3, 90.0, 88.5, 75.2, 69.9, 69.1, 68.5, 67.7, 66.7, 61.8, 60.0, 59.8, 42.1, 40.0, 39.7, 27.6, 13.9.

**IR** (neat): 2974, 1733, 1492, 1444, 1250, 1183, 1068, 760, 706, 693 cm^-1^.

**HRMS**: m/z calcd for C_46_H_45_FeNO_5_ [M+H]^+^ : 748.2720, found: 748.2727.

**mp**: 188‒190 ºC.

**Preparation of compound 3f**

Tetrayne **1d** (1.0 mmol), compound **2c** (1.1 mmol), were mixed in an oven-dried Schlenk tube (50 mL) equipped with a magnetic stir bar and heated in a 110 ºC oil bath in 10 mL toluene for 8 hours under argon atmosphere. Then the reaction mixture was cooled to room temperature, quenched with saturated NaCl, and extracted with ethyl acetate (3 × 10 mL). The combined organic extracts were dried over anhydrous MgSO_4_, filtered, and concentrated under reduced pressure. The crude product was purified by column chromatography on neutral aluminum oxide (petroleum ether : EtOAc = 40:1) to yield the product **3f** (513 mg, 70%) as an orange solid.

**Data for 3f**

**^1^H NMR** (500 MHz, C_6_D_6_): δ 7.84 (d, *J* = 7.7 Hz, 2H), 7.31-7.27 (m, 4H), 7.20 (d, *J* = 7.3 Hz, 1H), 6.95-6.89 (m, 3H), 6.80 (s, 1H), 4.86 (s, 1H), 4.80 (d, *J* = 2.1 Hz, 1H), 4.36-4.27 (m, 3H), 4.07 (dd, *J* = 11.4, 4.1 Hz, 1H), 4.02 (d, *J* = 1.4 Hz, 2H), 3.98 (s, 5H), 3.96-3.87 (m, 4H), 3.80 (d, *J* = 6.4 Hz, 2H), 3.46 (s, 1H), 0.90 (t, *J* = 7.1 Hz, 3H), 0.88 (t, *J* = 7.0 Hz, 3H), 0.77-0.76 (m, 6H).

**^13^C NMR** (125 MHz, C_6_D_6_): δ 171.7, 171.6, 171.6, 145.9, 144.9, 144.5, 142.2, 131.5, 129.9, 128.4, 128.3, 127.6, 127.4, 124.9, 123.6, 110.9, 107.0, 94.2, 89.1, 71.7, 71.6, 70.6, 70.6, 70.0, 64.9, 61.8, 60.1, 57.7, 42.1, 38.4, 30.6, 19.0, 18.5, 14.0, 13.9.

**IR** (neat): 2961, 1732, 1593, 1351, 1275, 1250, 1132, 768, 692 cm^-1^.

**HRMS**: m/z calcd for C_45_H_43_FeNO_5_ [M+H]^+^ : 734.2563, found: 734.2571

**mp**: 182‒184 ºC.

**Preparation of compound 3g**

Tetrayne **1e** (1.0 mmol), compound **2a** (1.1 mmol), were mixed in an oven-dried Schlenk tube (50 mL) equipped with a magnetic stir bar and heated in a 110 ºC oil bath in 10 mL toluene for 8 hours under argon atmosphere. Then the reaction mixture was cooled to room temperature, quenched with saturated NaCl, and extracted with ethyl acetate (3 × 10 mL). The combined organic extracts were dried over anhydrous MgSO_4_, filtered, and concentrated under reduced pressure. The crude product was purified by column chromatography on neutral aluminum oxide (petroleum ether : EtOAc = 40:1) to yield the product **3g** (565 mg, 73%) as an orange solid.

**Data for 3g**

**^1^H NMR** (500 MHz, C_6_D_6_): δ 7.78 (d, *J* = 7.8 Hz, 1H), 7.61 (d, *J* = 7.8 Hz, 1H), 7.22 (d, *J* = 7.5 Hz, 2H), 7.19 (s, 1H), 7.13 (d, *J* = 7.7 Hz, 1H), 6.78 (d, *J* = 7.6 Hz, 2H), 6.44 (s, 1H), 4.57 (d, *J* = 17.5 Hz, 1H), 4.49 (s, 1H), 4.35 (d, *J* = 17.4 Hz, 1H), 4.28 (d, *J* = 17.4 Hz, 1H), 4.09 (d, *J* = 11.9 Hz, 1H), 4.01 (d, *J* = 17.8 Hz, 1H), 3.96-3.87 (m, 5H), 3.76-3.73 (m, 3H), 3.63 (s, 5H), 2.15 (s, 3H), 1.96 (s, 3H), 1.02 (s, 9H), 0.87 (t, *J* = 5.7 Hz, 3H), 0.86 (t, *J* = 5.4 Hz, 3H).

**^13^C NMR** (125 MHz, C_6_D_6_): δ 179.5, 171.8, 171.7, 146.6, 144.5, 144.1, 138.1, 137.4, 136.8, 135.3, 132.0, 131.7, 130.4, 129.3, 129.2, 128.7, 128.7, 128.3, 121.2, 117.4, 97.5, 90.1, 88.1, 75.3, 69.9, 69.1, 68.6, 67.7, 66.7, 61.7, 60.0, 59.8, 42.2, 40.0, 39.7, 27.6, 21.3, 21.2, 13.9.

**IR** (neat): 2980, 1731, 1594, 1365, 1273, 1246, 1181, 1069, 819 cm^-1^.

**HRMS**: m/z calcd for C_48_H_49_FeNO_5_ [M+H]^+^ : 776.3033, found: 776.3039.

**mp**: 199‒202 ºC.

**Preparation of compound 3h**

Tetrayne **1f** (1.0 mmol), compound **2a** (1.1 mmol), were mixed in an oven-dried Schlenk tube (50 mL) equipped with a magnetic stir bar and heated in a 110 ºC oil bath in 10 mL toluene for 8 hours under argon atmosphere. Then the reaction mixture was cooled to room temperature, quenched with saturated NaCl, and extracted with ethyl acetate (3 × 10 mL). The combined organic extracts were dried over anhydrous MgSO_4_, filtered, and concentrated under reduced pressure. The crude product was purified by column chromatography on neutral aluminum oxide (petroleum ether : EtOAc = 40:1) to yield the product **3h** (524 mg, 65%) as an orange solid.

**Data for 3h**

**^1^H NMR** (500 MHz, C_6_D_6_): δ 7.82 (dd, *J* = 8.4, 1.8 Hz, 1H), 7.64 (dd, *J* = 8.5, 2.1 Hz, 1H), 7.24 (d, *J* = 8.5 Hz, 2H), 7.00 (dd, *J* = 8.4, 2.6 Hz, 1H), 6.90 (dd, *J* = 8.5, 2.5 Hz, 1H), 6.54 (d, *J* = 8.7 Hz, 2H), 6.48 (s, 1H), 4.59 (d, *J* = 17.4 Hz, 1H), 4.50 (s, 1H), 4.37 (d, *J* = 17.4 Hz, 1H), 4.31 (d, *J* = 17.4 Hz, 1H), 4.10 (d, *J* = 11.9 Hz, 1H), 4.03 (d, *J* = 17.4 Hz, 1H), 3.99-3.88 (m, 5H), 3.76-3.73 (m, 3H), 3.65 (s, 5H), 3.31 (s, 3H), 3.16 (s, 3H), 1.03 (s, 9H), 0.87 (t, *J* = 6.9 Hz, 3H), 0.86 (t, *J* = 7.0 Hz, 3H).

**^13^C NMR** (125 MHz, C_6_D_6_): δ 179.4, 171.9, 171.7, 160.0, 159.8, 146.5, 144.1, 143.9, 135.2, 133.3, 133.3, 132.0, 131.8, 128.8, 117.8, 116.3, 114.3, 113.8, 113.6, 97.5, 90.0, 87.5, 75.3, 70.0, 69.1, 68.6, 67.8, 66.7, 61.7, 59.9, 59.8, 54.9, 54.7, 42.3, 40.0, 39.7, 27.6, 13.9.

**IR** (neat): 2978, 1734, 1604, 1510, 1288, 1247, 1181, 1068, 837 cm^-1^.

**HRMS**: m/z calcd for C_48_H_49_FeNO_7_ [M+H]^+^ : 808.2931, found: 808.2938.

**mp**: 193‒196 ºC.

**Preparation of compound 3i**

Tetrayne **1g** (1.0 mmol), compound **2a** (1.1 mmol), were mixed in an oven-dried Schlenk tube (50 mL) equipped with a magnetic stir bar and heated in a 110 ºC oil bath in 10 mL toluene for 8 hours under argon atmosphere. Then the reaction mixture was cooled to room temperature, quenched with saturated NaCl, and extracted with ethyl acetate (3 × 10 mL). The combined organic extracts were dried over anhydrous MgSO_4_, filtered, and concentrated under reduced pressure. The crude product was purified by column chromatography on neutral aluminum oxide (petroleum ether : EtOAc = 40:1) to yield the product **3i** (660 mg, 81%) as an orange solid.

**Data for 3i**

**^1^H NMR** (400 MHz, C_6_D_6_): δ 7.51 (dd, *J* = 8.2, 2.1 Hz, 1H), 7.39 (dd, *J* = 8.2, 2.1 Hz, 1H), 7.27 (dd, *J* = 8.2, 2.2 Hz, 1H), 7.21 (dd, *J* = 8.2, 2.2 Hz, 1H), 6.87 (s, 4H), 6.24 (s, 1H), 4.50 (d, *J* = 17.5 Hz, 1H), 4.44 (s, 1H), 4.31 (d, *J* = 17.5 Hz, 1H), 4.19 (d, *J* = 17.5 Hz, 1H), 4.07-4.02 (m, 2H), 3.97 (d, *J* = 3.6 Hz, 1H), 3.96-3.95 (m, 1H), 3.92-3.90 (m, 1H), 3.88-3.83 (m, 2H), 3.71-3.65 (m, 3H), 3.59 (s, 5H), 1.00 (s, 9H), 0.87 (t, *J* = 8.6 Hz, 3H), 0.86 (t, *J* = 8.6 Hz, 3H).

**^13^C NMR** (125 MHz, C_6_D_6_): δ 180.0, 171.7, 171.6, 146.9, 144.3, 143.1, 137.9, 135.8, 134.4, 134.1, 133.3, 132.8, 131.9, 128.9, 128.3, 122.1, 116.7, 96.3, 89.5, 88.9, 75.2, 69.9, 69.1, 68.4, 67.9, 66.8, 61.9, 60.1, 59.7, 42.1, 39.9, 39.7, 27.5, 13.9, 13.9.

**IR** (neat): 2979, 1736, 1601, 1365, 1246, 1092, 1069, 832, 810 cm^-1^.

**HRMS**: m/z calcd for C_46_H_43_Cl_2_FeNO_5_ [M+H]^+^ : 816.1940, found: 816.1934.

**mp**: 191‒193 ºC.

**Preparation of compound 3j**

Tetrayne **1h** (1.0 mmol), compound **2a** (1.1 mmol), were mixed in an oven-dried Schlenk tube (50 mL) equipped with a magnetic stir bar and heated in a 110 ºC oil bath in 10 mL toluene for 8 hours under argon atmosphere. Then the reaction mixture was cooled to room temperature, quenched with saturated NaCl, and extracted with ethyl acetate (3 × 10 mL). The combined organic extracts were dried over anhydrous MgSO_4_, filtered, and concentrated under reduced pressure. The crude product was purified by column chromatography on neutral aluminum oxide (petroleum ether : EtOAc = 40:1) to yield the product **3j** (554 mg, 69%) as an orange solid.

**Data for 3j**

**^1^H NMR** (500 MHz, C_6_D_6_): δ 7.78 (d, *J* = 8.0 Hz, 1H), 7.61 (d, *J* = 7.9 Hz, 1H), 7.22 (d, *J* = 7.9 Hz, 3H), 7.12 (d, *J* = 7.7 Hz, 1H), 6.77 (d, *J* = 7.9 Hz, 2H), 6.45 (s, 1H), 4.99 (dq, *J* = 12.5, 6.2 Hz, 2H), 4.59 (d, *J* = 17.5 Hz, 1H), 4.49 (s, 1H), 4.33 (d, *J* = 13.5 Hz, 1H), 4.30 (d, *J* = 13.6 Hz, 1H), 4.08 (d, *J* = 11.9 Hz, 1H), 4.04 (d, *J* = 17.4 Hz, 1H), 3.88 (s, 1H), 3.75 (s, 1H), 3.72 (d, *J* = 6.7 Hz, 2H), 3.62 (s, 5H), 2.14 (s, 3H), 1.95 (s, 3H), 1.02 (s, 9H), 0.99-0.93 (m, 12H).

**^13^C NMR** (125 MHz, C_6_D_6_): δ 179.5, 171.4, 171.3, 146.7, 144.5, 144.2, 138.1, 137.3, 136.9, 135.3, 132.0, 131.7, 130.4, 129.3, 129.1, 128.7, 128.3, 121.3, 117.4, 97.4, 90.1, 88.1, 75.3, 69.9, 69.1, 68.6, 67.7, 66.7, 59.9, 59.9, 42.2, 40.0, 39.7, 27.6, 21.4, 21.3, 21.2.

**IR** (neat): 2980, 1726, 1600, 1365, 1253, 1183, 1104, 819, 776 cm^-1^.

**HRMS**: m/z calcd for C_50_H_53_FeNO_5_ [M+H]^+^ : 804.3346, found: 804.3353.

**mp**: 208‒210 ºC.

**Preparation of compound 4a**

Tetrayne **1a** (2.0 mmol), compound **2d** (1.0 mmol) and H_2_O (1.0 mmol) were mixed in an oven-dried Schlenk tube (50 mL) equipped with a magnetic stir bar and heated in a 105 ºC oil bath in 10 mL toluene for 12 hours under air atmosphere. Then the reaction mixture was cooled to room temperature, quenched with saturated NaCl, and extracted with ethyl acetate (3 × 10 mL). The combined organic extracts were dried over anhydrous MgSO_4_, filtered, and concentrated under reduced pressure. The crude product was purified by column chromatography on silica gel (petroleum ether : EtOAc = 60:1) to yield the product **4a** (608 mg, 81%) as an orange solid.

**Data for 4a**

**^1^H NMR** (500 MHz, C_6_D_6_): δ 8.73 (s, 1H), 7.74 (d, *J* = 7.7 Hz, 2H), 7.25-7.20 (m, 5H), 7.09-7.07 (m, 2H), 6.96 – 6.95 (m, 3H), 6.93-6.91 (m, 3H), 4.78 (s, 2H), 4.72 (s, 2H), 4.44 (s, 2H), 3.71 (s, 5H), 3.71 (s, 2H), 3.49 (t, *J* = 1.5 Hz, 2H), 3.30 (s, 6H).

**^13^C NMR** (125 MHz, C_6_D_6_): δ 172.4, 153.1, 147.4, 141.2, 139.4, 138.4, 132.0, 131.8, 129.9, 128.7, 128.5, 128.3, 127.0, 126.5, 126.5, 124.1, 123.3, 120.5, 97.4, 89.3, 80.8, 71.5, 69.5, 68.7, 59.7, 52.5, 42.9, 41.3.

**IR** (neat): 3453, 2813, 1734, 1519, 1386, 1350, 1264, 754, 695 cm^-1^.

**HRMS**: m/z calcd for C_47_H_37_FeNO_5_ [M+H]^+^ : 752.2094, found: 752.2098.

**mp**: 214‒217 ºC.

**Preparation of compound 4b**

Tetrayne **1i** (2.0 mmol), compound **2d** (1.0 mmol) and H_2_O (1.0 mmol) were mixed in an oven-dried Schlenk tube (50 mL) equipped with a magnetic stir bar and heated in a 105 ºC oil bath in 10 mL toluene for 12 hours under air atmosphere. Then the reaction mixture was cooled to room temperature, quenched with saturated NaCl, and extracted with ethyl acetate (3 × 10 mL). The combined organic extracts were dried over anhydrous MgSO_4_, filtered, and concentrated under reduced pressure. The crude product was purified by column chromatography on silica gel (petroleum ether : EtOAc = 60:1) to yield the product **4b** (599 mg, 77%) as an orange solid.

**Data for 4b**

**^1^H NMR** (500 MHz, C_6_D_6_): δ 8.73 (s, 1H), 7.75 (d, *J* = 7.6 Hz, 2H), 7.25-7.20 (m, 5H), 7.02 (d, *J* = 8.1 Hz, 2H), 6.78 (t, *J* = 7.5 Hz, 4H), 4.78 (s, 2H), 4.73 (s, 2H), 4.46 (s, 2H), 3.72 (s, 5H), 3.72 (d, *J* = 1.5 Hz, 2H), 3.47 (t, *J* = 1.7 Hz, 2H), 3.29 (s, 6H), 2.16 (s, 3H), 1.94 (s, 3H).

**^13^C NMR** (125 MHz, C_6_D_6_): δ 172.4, 152.9, 147.4, 141.2, 139.9, 139.5, 138.2, 135.6, 131.9, 131.8, 129.9, 129.3, 128.7, 128.3, 126.5, 123.3, 121.3, 120.6, 97.6, 88.9, 81.0, 71.5, 69.5, 68.5, 59.7, 52.5, 42.9, 41.4, 41.4, 21.3.

**IR** (neat): 3449, 2811, 1736, 1590, 1511, 1386, 1350, 1271, 819, 697 cm^-1^.

**HRMS**: m/z calcd for C_49_H_41_FeNO_5_ [M+H]^+^ : 780.2407, found: 780.2410.

**mp**: 219‒222 ºC.

**Preparation of compound 4c**

Tetrayne **1d** (2.0 mmol), compound **2d** (1.0 mmol) and H_2_O (1.0 mmol) were mixed in an oven-dried Schlenk tube (50 mL) equipped with a magnetic stir bar and heated in a 105 ºC oil bath in 10 mL toluene for 12 hours under air atmosphere. Then the reaction mixture was cooled to room temperature, quenched with saturated NaCl, and extracted with ethyl acetate (3 × 10 mL). The combined organic extracts were dried over anhydrous MgSO_4_, filtered, and concentrated under reduced pressure. The crude product was purified by column chromatography on silica gel (petroleum ether : EtOAc = 60:1) to yield the product **4c** (576 mg, 74%) as an orange solid.

**Data for 4c**

**^1^H NMR** (500 MHz, C_6_D_6_): δ 8.73 (s, 1H), 7.74 (d, *J* = 7.7 Hz, 2H), 7.24-7.19 (m, 4H), 7.08-7.03 (m, 3H), 6.96-6.94 (m, 3H), 6.94-6.91 (m, 3H), 4.83 (s, 2H), 4.72 (s, 2H), 4.49 (s, 2H), 3.97 (q, *J* = 7.0 Hz, 4H), 3.71 (s, 5H), 3.70-3.69 (m, 2H), 3.48 (d, *J* = 1.7 Hz, 2H), 0.90 (t, *J* = 6.9 Hz, 6H).

**^13^C NMR** (125 MHz, C_6_D_6_): δ 172.0, 153.0, 147.4, 141.2, 141.2, 141.0, 139.9, 139.6, 138.5, 132.0, 131.8, 129.9, 128.7, 128.5, 128.3, 127.1, 127.0, 126.5, 126.4, 124.1, 123.2, 120.5, 97.3, 89.3, 80.8, 71.5, 69.5, 68.7, 61.7, 59.9, 42.8, 41.4, 41.3, 14.0.

**IR** (neat): 3445, 2986, 1731, 1662, 1591, 1448, 1282, 1244, 780, 759, 702 cm^-1^.

**HRMS**: m/z calcd for C_49_H_41_FeNO_5_ [M+H]^+^ : 780.2407, found: 780.2410.

**mp**: 208‒212 ºC.

**Preparation of compound 4d**

Tetrayne **1j** (2.0 mmol), compound **2d** (1.0 mmol) and H_2_O (1.0 mmol) were mixed in an oven-dried Schlenk tube (50 mL) equipped with a magnetic stir bar and heated in a 105 ºC oil bath in 10 mL toluene for 12 hours under air atmosphere. Then the reaction mixture was cooled to room temperature, quenched with saturated NaCl, and extracted with ethyl acetate (3 × 10 mL). The combined organic extracts were dried over anhydrous MgSO_4_, filtered, and concentrated under reduced pressure. The crude product was purified by column chromatography on silica gel (petroleum ether : EtOAc = 60:1) to yield the product **4d** (572 mg, 71%) as an orange solid.

**Data for 4d**

**^1^H NMR** (500 MHz, C_6_D_6_): δ 8.72 (s, 1H), 7.74 (d, *J* = 7.4 Hz, 2H), 7.24-7.20 (m, 4H), 7.07-7.04 (m, 3H), 6.96-6.89 (m, 6H), 5.08-5.01 (m, 2H), 4.83 (s, 2H), 4.71 (s, 2H), 4.50 (s, 2H), 3.71 (s, 5H), 3.70-3.69 (m, 2H), 3.49-3.48 (m, 2H), 1.02 (d, *J* = 6.3 Hz, 6H), 1.00 (d, *J* = 6.1 Hz, 6H).

**^13^C NMR** (125 MHz, C_6_D_6_): δ 171.5, 153.0, 147.4, 141.2, 141.1, 139.9, 139.7, 138.6, 132.0, 131.8, 129.9, 128.6, 128.4, 128.3, 127.0, 126.5, 126.4, 124.2, 123.2, 120.5, 97.3, 89.4, 80.8, 71.5, 69.5, 69.0, 68.7, 60.0, 42.7, 41.4, 21.5, 21.4.

**IR** (neat): 3447, 2809, 1732, 1586, 1387, 1259, 1106, 753, 696 cm^-1^.

**HRMS**: m/z calcd for C_51_H_45_FeNO_5_ [M+H]^+^ : 808.2720, found: 808.2725.

**mp**: 204‒208 ºC.

**Preparation of compound 4e**

Tetrayne **1a** (2.0 mmol), compound **2c** (1.0 mmol) and H_2_O (1.0 mmol) were mixed in an oven-dried Schlenk tube (50 mL) equipped with a magnetic stir bar and heated in a 105 ºC oil bath in 10 mL toluene for 12 hours under air atmosphere. Then the reaction mixture was cooled to room temperature, quenched with saturated NaCl, and extracted with ethyl acetate (3 × 10 mL). The combined organic extracts were dried over anhydrous MgSO_4_, filtered, and concentrated under reduced pressure. The crude product was purified by column chromatography on silica gel (petroleum ether : EtOAc = 60:1) to yield the product **4e** (534 mg, 76%) as an orange solid.

**Data for 4e**

**^1^H NMR** (500 MHz, CDCl_3_): δ 8.67 (s, 1H), 7.24-7.21 (m, 5H), 7.04 (s, 5H), 4.23 (s, 2H), 4.11 (s, 5H), 4.00 (s, 2H), 3.98 (s, 2H), 3.93 (s, 2H), 3.82 (s, 6H), 3.74-3.68 (m, 1H), 1.48 (s, 6H).

**^13^C NMR** (101 MHz, CDCl_3_): δ 172.6, 158.0, 145.9, 140.5, 139.7, 138.1, 137.2, 131.3, 128.2, 126.7, 125.9, 123.6, 121.4, 120.8, 118.9, 96.1, 88.2, 80.1, 71.2, 69.3, 68.5, 58.9, 53.1, 42.1, 40.4, 30.8, 21.2.

**IR** (neat): 3243, 2959, 2922, 1730, 1435, 1372, 1252, 1199, 1157, 1074, 756, 692 cm^-1^.

**HRMS**: *m/z* calcd for C_43_H_37_FeNO_5_ [M+H]^+^: 704.2094, found: 704.2072.

**mp**: 167‒169 ºC.

**Preparation of compound 4f**

Tetrayne **1e** (2.0 mmol), compound **2c** (1.0 mmol) and H_2_O (1.0 mmol) were mixed in an oven-dried Schlenk tube (50 mL) equipped with a magnetic stir bar and heated in a 105 ºC oil bath in 10 mL toluene for 12 hours under air atmosphere. Then the reaction mixture was cooled to room temperature, quenched with saturated NaCl, and extracted with ethyl acetate (3 × 10 mL). The combined organic extracts were dried over anhydrous MgSO_4_, filtered, and concentrated under reduced pressure. The crude product was purified by column chromatography on silica gel (petroleum ether : EtOAc = 60:1) to yield the product **4f** (538 mg, 71%) as an orange solid.

**Data for 4f**

**^1^H NMR** (500 MHz, CDCl_3_): δ 8.64(s, 1H), 7.15 (d, *J* = 8.0 Hz, 2H), 7.06 (d, *J* = 7.5 Hz, 2H), 6.93 (d, *J* = 7.5 Hz, 2H), 6.82 (d, *J* = 7.5 Hz, 2H), 4.28 (q, *J* = 7.0 Hz, 4H), 4.20 (s, 2H), 4.10 (s, 5H), 3.98 (s, 2H), 3.96 (s, 2H), 3.94 (s, 2H), 3.70-3.68 (m, 1H), 2.32 (s, 3H), 2.25 (s, 3H), 1.48 (s, 3H), 1.46 (s, 3H), 1.32 (t, *J* = 7.0 Hz, 6H).

**^13^C NMR** (101 MHz, CDCl_3_): δ 172.3, 157.6, 146.1, 140.3, 140.0, 138.3, 138.1, 137.4, 136.9, 135.6, 131.2, 128.8, 127.4, 126.2, 121.9, 120.7, 119.3, 96.3, 88.2, 80.7, 71.2, 69.2, 68.4, 61.8, 59.1, 42.1, 40.4, 30.9, 29.7, 21.4, 21.2, 14.1.

**IR** (neat): 3251, 2959, 2919, 1735, 1512, 1374, 1247, 1074, 819, 591 cm^-1^.

**HRMS**: *m/z* calcd for C_47_H_45_FeNO_5_ [M+H]^+^: 760.2720, found: 760.2729.

**mp**: 192‒194 ºC.

**Preparation of compound 4g**

Tetrayne **1k** (2.0 mmol), compound **2c** (1.0 mmol) and H_2_O (1.0 mmol) were mixed in an oven-dried Schlenk tube (50 mL) equipped with a magnetic stir bar and heated in a 105 ºC oil bath in 10 mL toluene for 12 hours under air atmosphere. Then the reaction mixture was cooled to room temperature, quenched with saturated NaCl, and extracted with ethyl acetate (3 × 10 mL). The combined organic extracts were dried over anhydrous MgSO_4_, filtered, and concentrated under reduced pressure. The crude product was purified by column chromatography on silica gel (petroleum ether : EtOAc = 60:1) to yield the product **4g** (638 mg, 81%) as an orange solid.

**Data for 4g**

**^1^H NMR** (400 MHz, CDCl_3_): δ 8.65 (s, 1H), 7.15 (d, *J* = 8.0 Hz, 2H), 7.07 (d, *J* = 8.0 Hz, 2H), 6.95 (d, *J* = 7.8 Hz, 2H), 6.84 (d, *J* = 7.8 Hz, 2H), 4.28 (q, *J* = 7.1 Hz, 4H), 4.20 (s, 2H), 4.09 (s, 5H), 3.97 (d, *J* = 6.2 Hz, 4H), 3.91 (s, 2H), 3.75-3.63 (m, 1H), 2.60 (q, *J* = 7.7 Hz, 2H), 2.55 (q, *J* = 7.7 Hz, 2H), 1.46 (d, *J* = 6.5 Hz, 6H), 1.32 (t, *J* = 7.1 Hz, 6H), 1.21 (t, *J* = 7.4 Hz, 3H), 1.20 (t, *J* = 7.4 Hz, 3H).

**^13^C NMR** (101 MHz, CDCl_3_): δ 172.3, 157.6, 146.1, 144.4, 142.1, 140.4, 140.0, 138.3, 137.8, 136.9, 131.4, 131.3, 127.7, 126.3, 126.2, 121.9, 120.9, 119.3, 96.4, 88.2, 80.6, 71.2, 69.2, 68.5, 61.8, 59.1, 42.1, 40.4, 30.9, 28.8, 21.2, 16.3, 15.4, 14.1.

**IR** (neat): 3257, 2967, 2930, 1738, 1512, 1372, 1250, 1175, 1074, 833, 532, cm^-1^.

**HRMS**: *m/z* calcd for C_49_H_49_FeNO_5_ [M+H]^+^: 788.3033, found: 788.3024.

**mp**: 165‒167 ºC.

**Preparation of compound 4h**

Tetrayne **1d** (2.0 mmol), compound **2c** (1.0 mmol) and H_2_O (1.0 mmol) were mixed in an oven-dried Schlenk tube (50 mL) equipped with a magnetic stir bar and heated in a 105 ºC oil bath in 10 mL toluene for 12 hours under air atmosphere. Then the reaction mixture was cooled to room temperature, quenched with saturated NaCl, and extracted with ethyl acetate (3 × 10 mL). The combined organic extracts were dried over anhydrous MgSO_4_, filtered, and concentrated under reduced pressure. The crude product was purified by column chromatography on silica gel (petroleum ether : EtOAc = 60:1) to yield the product **4h** (570 mg, 78%) as an orange solid.

**Data for 4h**

**^1^H NMR** (400 MHz, CDCl_3_): δ 8.65 (s, 1H), 7.24 (s, 5H), 7.04 (s, 5H), 4.28 (q, *J* = 6.9 Hz, 4H), 4.20 (s, 2H), 4.10 (s, 5H), 4.00 (s, 2H), 3.97 (s, 2H), 3.93 (s, 2H), 3.73-3.64 (m, 1H), 1.47 (d, *J* = 5.8 Hz, 6H), 1.32 (t, *J* = 6.9 Hz, 6H).

**^13^C NMR** (101 MHz, CDCl_3_): δ 172.2, 157.8, 146.1, 140.5, 140.4, 140.1, 138.4, 137.2, 131.4, 131.3, 128.2, 128.0, 126.7, 126.2, 126.1, 123.6, 121.9, 119.2, 96.1, 88.7, 80.6, 71.2, 69.2, 68.6, 61.8, 59.1, 42.1, 40.3, 30.9, 21.2, 14.1.

**IR** (neat): 3251, 2959, 2856, 1733, 1605, 1515, 1372, 1252, 1072, 830, 538 cm^-1^.

**HRMS**: *m/z* calcd for C_45_H_41_FeNO_5_ [M+H]^+^: 732.2407, found: 732.2412.

**mp**: 175‒177 ºC.

**Preparation of compound 4i**

Tetrayne **1k** (2.0 mmol), compound **2c** (1.0 mmol) and H_2_O (1.0 mmol) were mixed in an oven-dried Schlenk tube (50 mL) equipped with a magnetic stir bar and heated in a 105 ºC oil bath in 10 mL toluene for 12 hours under air atmosphere. Then the reaction mixture was cooled to room temperature, quenched with saturated NaCl, and extracted with ethyl acetate (3 × 10 mL). The combined organic extracts were dried over anhydrous MgSO_4_, filtered, and concentrated under reduced pressure. The crude product was purified by column chromatography on silica gel (petroleum ether : EtOAc = 60:1) to yield the product **4i** (523 mg, 69%) as an orange solid.

**Data for 4i**

**^1^H NMR** (500 MHz, C_6_D_6_): *δ* 8.87 (s, 1H), 7.23 (d, *J* = 7.7 Hz, 2H), 7.10 (dd, *J* = 5.7, 2.4 Hz, 2H), 7.00-6.96 (m, 3H), 6.95-6.89 (m, 3H), 5.07-4.99 (m, 2H), 4.78 (s, 2H), 4.49 (s, 2H), 4.05 (dt, *J* = 13.7, 6.8 Hz, 1H), 3.84 (s, 5H), 3.78 (s, 2H), 3.53 (s, 2H), 1.69 (d, *J* = 6.8 Hz, 6H), 1.00 (d, *J* = 6.2 Hz, 6H), 0.99 (d, *J* = 6.1 Hz, 6H).

**^13^C NMR** (125 MHz, C_6_D_6_): δ 171.6, 158.2, 146.7, 141.2, 141.1, 141.0, 139.4, 138.5, 132.0, 131.8, 128.4, 128.3, 127.0, 126.7, 126.4, 124.2, 122.2, 120.2, 97.1, 89.5, 81.1, 71.6, 69.5, 69.0, 68.7, 59.9, 42.8, 41.1, 31.5, 21.6, 21.5, 21.4.

**IR** (neat): 3255, 2981, 1728, 1587, 1563, 1280, 1253, 1106, 1053, 762, 704, 692 cm^-1^.

**HRMS**: *m/z* calcd for C_47_H_45_FeNO_5_ [M+H]^+^: 760.2720, found 760.2722.

**mp**: 198‒201 ºC.

**Preparation of compound 4j**

Tetrayne **1l** (2.0 mmol), compound **2c** (1.0 mmol) and H_2_O (1.0 mmol) were mixed in an oven-dried Schlenk tube (50 mL) equipped with a magnetic stir bar and heated in a 105 ºC oil bath in 10 mL toluene for 12 hours under air atmosphere. Then the reaction mixture was cooled to room temperature, quenched with saturated NaCl, and extracted with ethyl acetate (3 × 10 mL). The combined organic extracts were dried over anhydrous MgSO_4_, filtered, and concentrated under reduced pressure. The crude product was purified by column chromatography on silica gel (petroleum ether : EtOAc = 60:1) to yield the product **4j** (603 mg, 74%) as an orange solid.

**Data for 4j**

**^1^H NMR** (400 MHz, CDCl_3_): δ 8.65 (s, 1H), 7.14 (d, *J* = 8.0 Hz, 2H), 7.07 (d, *J* = 8.0 Hz, 2H), 6.95 (d, *J* = 7.8 Hz, 2H), 6.84 (d, *J* = 7.6 Hz, 2H), 5.17-5.05 (m, 2H), 4.16 (s, 2H), 4.10 (s, 5H), 3.98 (s, 2H), 3.92 (d, *J* = 5.8 Hz, 4H), 3.76-3.64 (m, 1H), 2.61 (q, *J* = 7.7 Hz, 2H), 2.55 (q, *J* = 7.9 Hz 2H), 1.48 (s, 6H), 1.30 (t, *J* = 5.6 Hz, 12H), 1.22 (d, *J* = 7.4 Hz, 3H), 1.19 (d, *J* = 7.5 Hz, 3H).

**^13^C NMR** (101 MHz, CDCl_3_): δ 171.8, 157.6, 146.0, 144.4, 142.1, 140.4, 140.0, 138.4, 137.8, 137.0, 131.4, 131.3, 127.7, 126.3, 126.2, 121.9, 121.0, 119.3, 96.3, 88.3, 80.7, 71.2, 69.2, 69.1, 68.5, 59.1, 42.1, 40.3, 30.9, 28.8, 21.6, 21.2, 16.3, 15.4.

**IR** (neat): 3251, 2970, 1733, 1507, 1462, 1374, 1252, 1175, 1098, 835, 535 cm^-1^.

**HRMS**: *m/z* calcd for C_51_H_53_FeNO_5_ [M+H]^+^: 816.3346, found 816.3340.

**mp**: 162‒164 ºC.

**Preparation of compound 4k**

Tetrayne **1c** (2.0 mmol), compound **2c** (1.0 mmol) and H_2_O (1.0 mmol) were mixed in an oven-dried Schlenk tube (50 mL) equipped with a magnetic stir bar and heated in a 105 ºC oil bath in 10 mL toluene for 12 hours under air atmosphere. Then the reaction mixture was cooled to room temperature, quenched with saturated NaCl, and extracted with ethyl acetate (3 × 10 mL). The combined organic extracts were dried over anhydrous MgSO_4_, filtered, and concentrated under reduced pressure. The crude product was purified by column chromatography on silica gel (petroleum ether : EtOAc = 60:1) to yield the product **4k** (532 mg, 73%) as an orange solid.

**Data for 4k**

**^1^H NMR** (500 MHz, C_6_D_6_): δ 8.72 (s, 1H), 7.84 (d, *J* = 8.0 Hz, 2H), 6.61 (d, *J* = 8.0 Hz, 2H), 5.53 (s, 2H), 5.23 (s, 2H), 4.13 (s, 2H), 4.03 (s, 2H), 3.96-3.91 (m, 1H), 3.90 (s, 5H), 2.96 (t, *J* = 7.8 Hz, 2H), 2.20 (t, *J* = 6.6 Hz, 2H), 1.76 (s, 3H), 1.58 (d, *J* = 6.8 Hz, 6H), 1.41-1.30 (m, 8H), 1.18-1.13 (m, 2H), 1.09-1.05 (m, 2H), 0.81 (t, *J* = 6.8 Hz, 3H), 0.73 (t, *J* = 7.2 Hz, 3H).

**^13^C NMR** (125 MHz, C_6_D_6_): δ 158.0, 146.3, 142.6, 142.5, 139.0, 136.2, 135.6, 132.8, 129.8, 128.3, 128.1, 127.9, 127.1, 121.1, 118.3, 99.1, 82.4, 78.5, 71.7, 70.0, 69.8, 56.3, 55.2, 34.2, 33.2, 31.4, 31.1, 23.2, 22.3, 21.4, 21.1, 19.6, 14.2, 13.7.

**IR** (neat): 3439, 2977, 2926, 2218, 1713, 1599, 1372, 1341, 1149, 1094, 822, 665, 604 cm^-1^.

**HRMS**: *m/z* calcd for C_43_H_50_FeN_2_O_3_S [M+H]^+^: 731.2964, found: 731.2957.

**mp**: 218‒220 ºC.

**Preparation of compound 5a**

Benzyne precursor **1m** (1.0 mmol), compound **2c** (1.0 mmol), 18-crown-6 (2 mmol) and CsF (2 mmol) are mixed in an oven-dried Schlenk tube (50 mL) equipped with a magnetic stir bar and heated in a 70 ºC oil bath in 5 mL toluene for 10 hours under air atmosphere. Then the reaction mixture was cooled to room temperature, quenched with saturated NaCl, and extracted with ethyl acetate (3 × 10 mL). The combined organic extracts were dried over anhydrous MgSO_4_, filtered, and concentrated under reduced pressure. The crude product was purified by column chromatography on silica gel (petroleum ether : EtOAc = 60:1) to yield the product **5a** (261 mg, 67%) as an orange solid.

**Data for 5a**

**^1^H NMR** (400 MHz, CDCl_3_): δ 7.18 (t, *J* = 7.9 Hz, 2H), 6.72-6.65 (m, 3H), 4.76 (d, *J* = 6.4 Hz, 2H), 4.38-4.37 (m, 2H), 4.37-4.34 (m, 1H), 4.22 (dd, *J* = 11.3, 4.9 Hz, 1H), 4.13 (s, 5H), 3.57 (dd, *J* = 10.7, 5.4 Hz, 1H), 2.09-2.05 (m, 1H), 1.09 (d, *J* = 7.0 Hz, 3H), 1.07 (d, *J* = 6.9 Hz, 3H).

**^13^C NMR** (101 MHz, CDCl_3_): δ 171.9, 147.84, 129.4, 117.4, 113.3, 71.4, 71.0, 70.2, 70.1, 69.8, 64.4, 57.5, 31.5, 30.1, 29.7, 19.3, 18.5.

**IR** (neat): 2962, 1696, 1523, 1499, 1281, 1141, 1024, 947, 819, 748, 695 cm^-1^.

**HRMS** (ESI-TOF): *m/z* calcd for C_22_H_25_FeNO_2_ [M+H]^+^: 392.1307, found 392.1302.

**mp**: 115‒117 ºC.

1. **X-Ray Crystallographic Data of 3a, 4d, and 5a.**

**
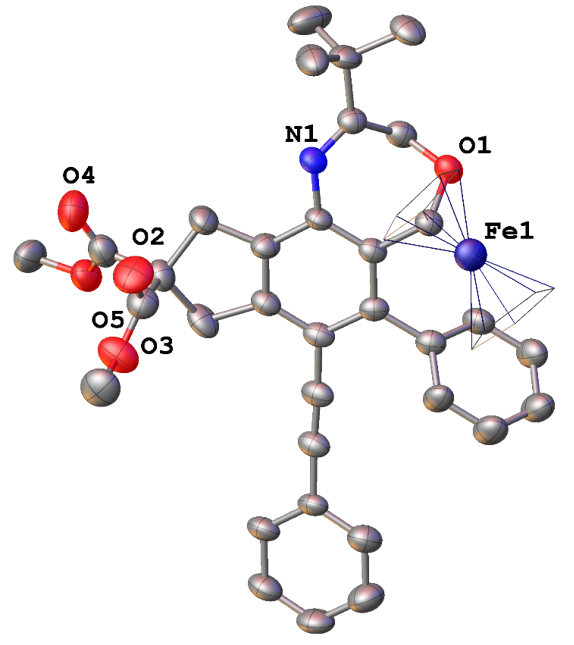
**

**Figure S1.** Molecular structure of **3a** showing thermal ellipsoid at the 30% probability level

**
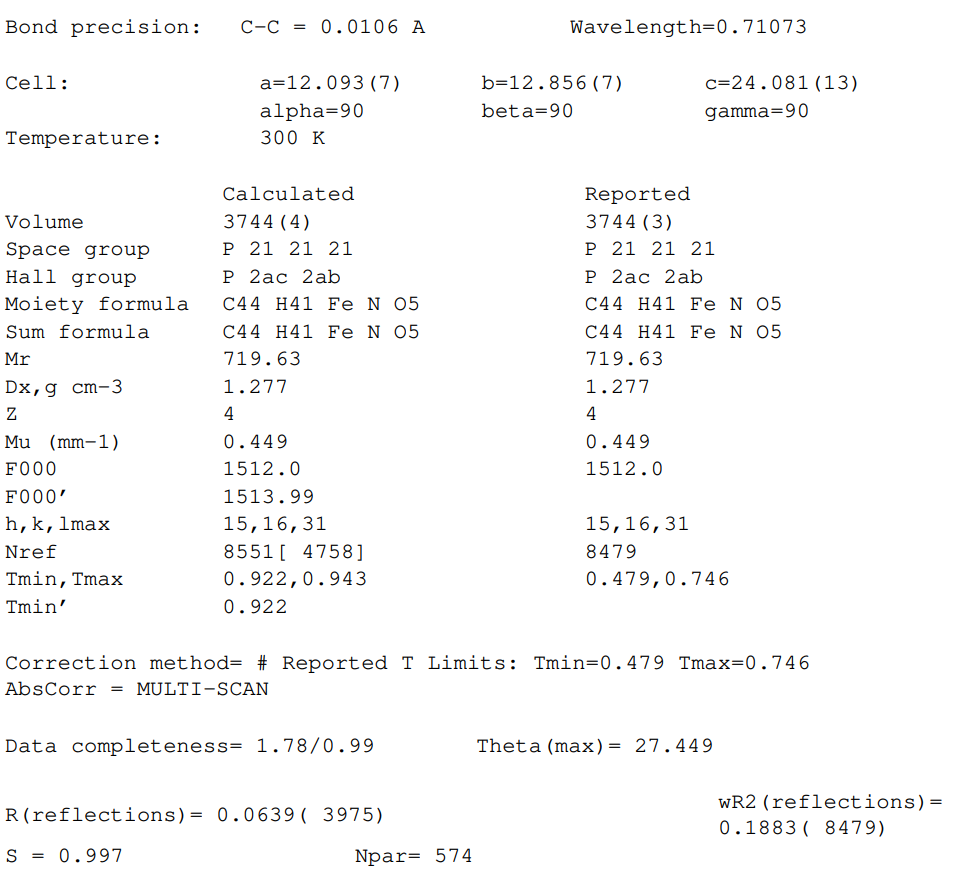
**


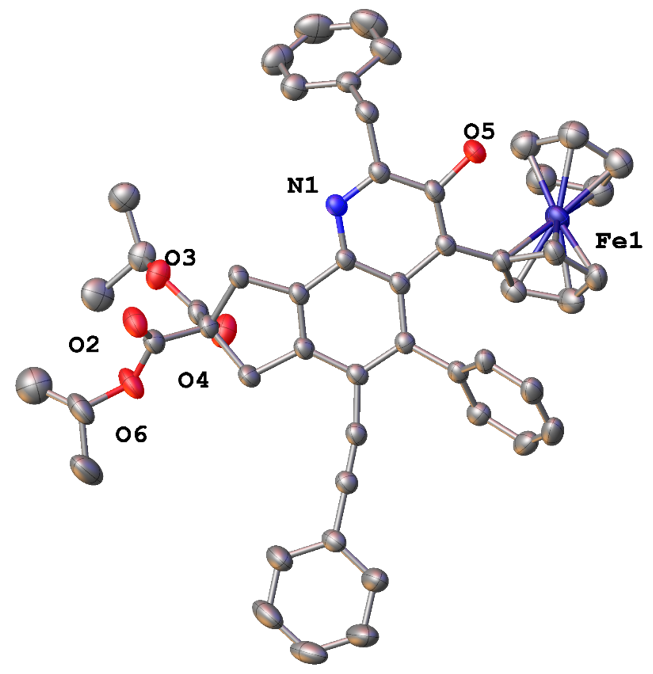


**Figure S2.** Molecular structure of **4d** showing thermal ellipsoid at the 30% probability level


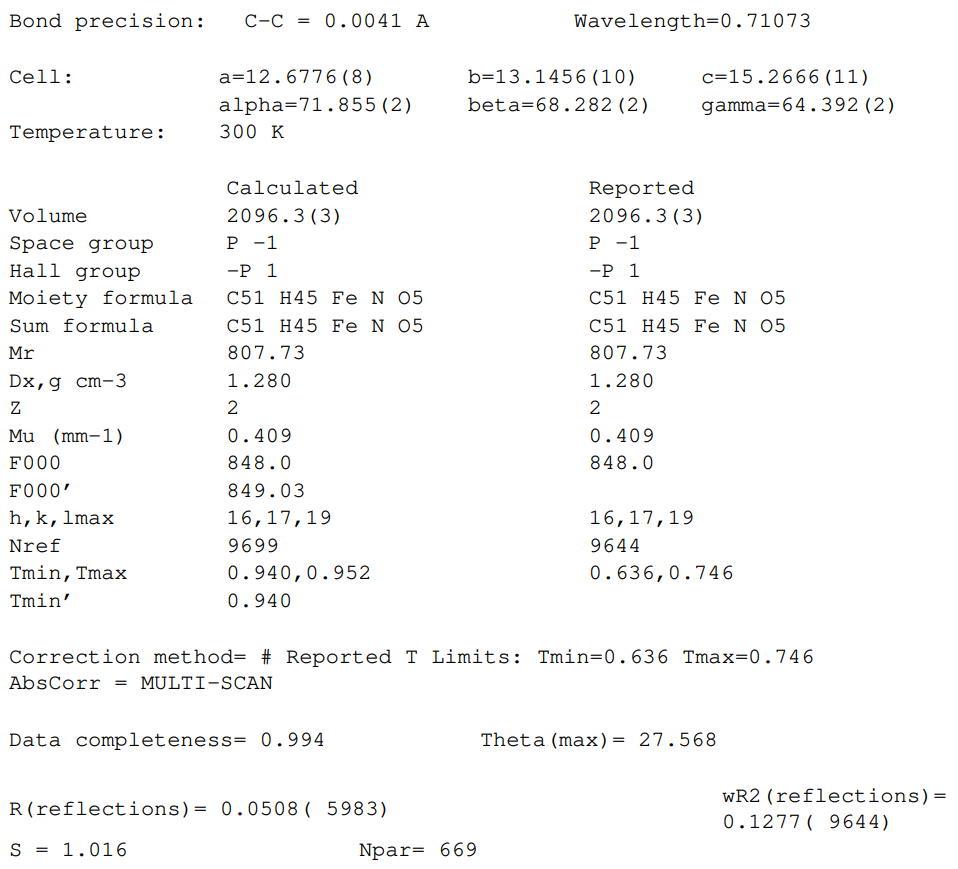


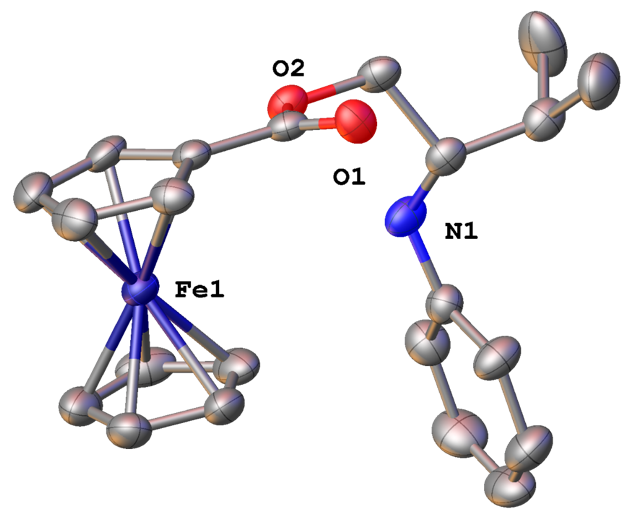


**Figure S3.** Molecular structure of **5a** showing thermal ellipsoid at the 30% probability level


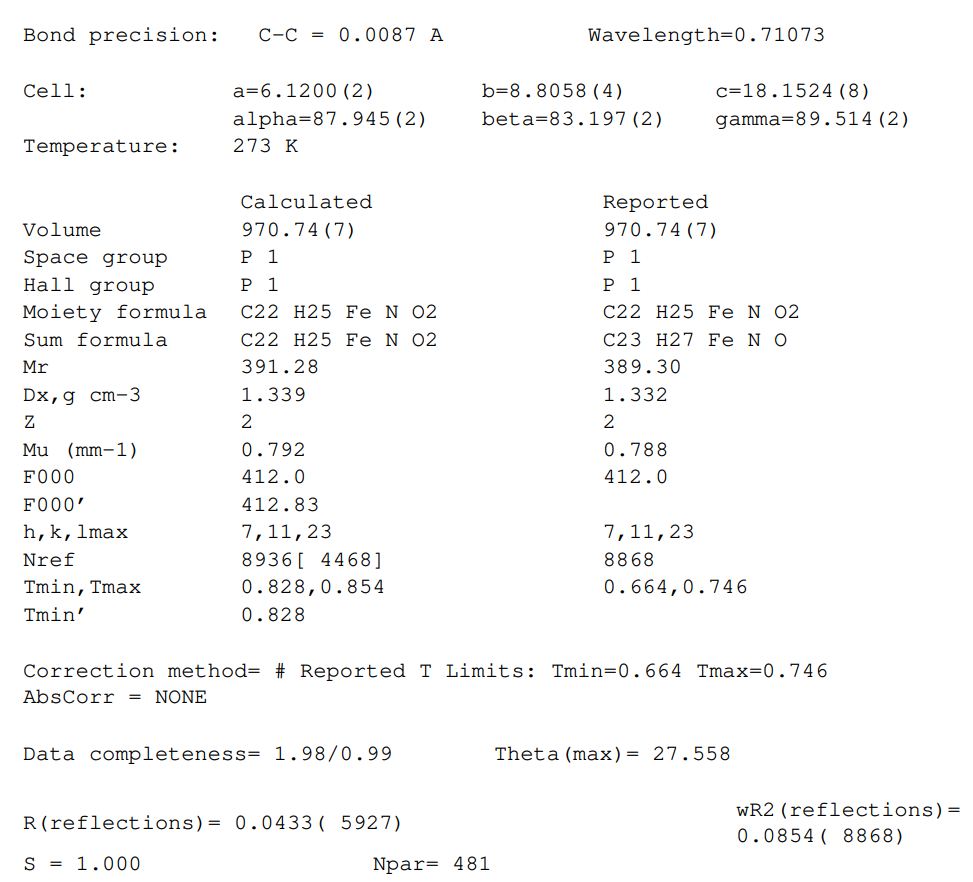


1. **Computational Details**

All calculations were performed by using gaussian09 program^1^. The geometry optimizations, frequency analysis and thermodynamic corrections were performed at the B3LYP(D3BJ)^2^//MDF10^3^ (for Cp_2_Fe)/6-311G**^4^ (for others) level of theory.

**References**

1. Frisch, M. J., Trucks, G. W., Schlegel, H. B., Scuseria, G. E., Robb, M. A., Cheeseman, J. R. et al. Gaussian 09, Revision E.01, Gaussian, Inc.: Wallingford, CT (2009).

2. Grimme, S., Ehrlich, S., and Goerigk, L. (2011). Effect of the damping function in dispersion corrected density functional theory. *J. Comput. Chem.* 32, 1456.

3. Dolg, M., Wedig, U., Stoll, H., and Preuss, H. (1987). Energy-adjusted *ab initio* pseudopotentials for the first row transition elements. *J. Chem. Phys.* 86, 866.

4. Francl, M. M., Pietro, W. J., Hehre, W. J., Binkley, J. S., Gordon, M. S., DeFrees, D. J. et al. (1982). Self-consistent molecular orbital methods. XXIII. a polarization-type basis set for second-row elements. *J. Chem. Phys.* 77, 3654‒3665.

**Free Energy and Geometry for 2a**

| Zero-point correction= | 0.343132 (Hartree/Particle) |
| --- | --- |
| Thermal correction to Energy= | 0.361744 |
| Thermal correction to Enthalpy= | 0.362689 |
| Thermal correction to Gibbs Free Energy= | 0.295116 |
| Sum of electronic and zero-point Energies= | -914.305027 |
| Sum of electronic and thermal Energies= | -914.286415 |
| Sum of electronic and thermal Enthalpies= | -914.285470 |
| Sum of electronic and thermal Free Energies= | -914.353043 |

Cartesian Coordinates:

C -0.82489800 0.29126200 -0.32760400

N -1.64880100 0.06523700 0.61571800

O -1.11114100 -0.20283500 -1.57068600

C -2.38790500 -0.87517800 -1.44658200

C -2.76003100 -0.71809100 0.05320100

C -4.13471100 -0.05970900 0.33801900

C -4.33730200 -0.01204500 1.86033700

C -5.24088900 -0.91783000 -0.29552900

C -4.18363500 1.36851500 -0.22396100

H -2.25482600 -1.91340700 -1.75182700

H -3.08822200 -0.38968200 -2.12712100

H -2.76960700 -1.69864800 0.54383600

H -5.29537500 0.45529800 2.10549300

H -3.53648900 0.55615400 2.33479100

H -4.33627300 -1.02029700 2.28621700

H -6.22547200 -0.50671900 -0.05685400

H -5.15811100 -0.95581100 -1.38564400

H -5.20732800 -1.94440200 0.08278700

H -3.40399500 1.98548600 0.22643500

H -4.04861900 1.38995300 -1.30911900

H -5.15240500 1.82597700 -0.00599500

C 0.42632300 1.02496900 -0.22261400

C 1.31823900 1.33839500 -1.29838400

C 1.01547400 1.49422500 0.99386700

C 2.44950300 1.99559500 -0.74392100

H 1.15621300 1.08663800 -2.33343700

C 2.26245500 2.09114400 0.66815600

H 0.58291500 1.37533500 1.97355600

Fe 2.23145300 0.10126700 0.07474400

H 3.31536400 2.33321100 -1.29173500

H 2.96355300 2.51251800 1.37155600

C 2.72848500 -1.65456100 -0.89136100

C 3.90366500 -1.02719900 -0.38294400

C 1.86337500 -1.92323800 0.20930200

H 2.51361600 -1.85512700 -1.92921700

C 3.76372500 -0.90840300 1.03055700

H 4.73715800 -0.67255600 -0.96906500

C 2.50230800 -1.46327600 1.39689600

H 0.87611500 -2.35468500 0.15009600

H 4.47326700 -0.44915300 1.70110000

H 2.08638700 -1.49445200 2.39167700

**Free Energy and Geometry for IN1**

| Zero-point correction= | 0.395377 (Hartree/Particle) |
| --- | --- |
| Thermal correction to Energy= | 0.423119 |
| Thermal correction to Enthalpy= | 0.424063 |
| Thermal correction to Gibbs Free Energy= | 0.332399 |
| Sum of electronic and zero-point Energies= | -1341.716020 |
| Sum of electronic and thermal Energies= | -1341.688278 |
| Sum of electronic and thermal Enthalpies= | -1341.687334 |
| Sum of electronic and thermal Free Energies= | -1341.778998 |

Cartesian Coordinates:

C 1.54987800 -2.27884500 -0.43005600

C 0.81712000 -1.07904300 -0.39629400

C -0.59531700 -1.02842200 -0.31339300

C -1.38099100 -2.23867000 -0.27351700

C -0.53085200 -3.33277400 -0.29183200

C 0.70799200 -3.37300400 -0.38188900

C 3.02691900 -2.04069900 -0.53121800

C 3.15297600 -0.53408000 -0.17254800

C 1.75829500 0.09499100 -0.48572600

C -1.21175800 0.24435300 -0.21548700

C -1.72072400 1.33712400 -0.11419900

C -2.39859800 2.57960300 -0.00722600

C -1.71880400 3.79629400 -0.18758100

C -2.40183400 5.00136900 -0.08744000

C -3.76744300 5.01500600 0.19264400

C -4.44991200 3.81287200 0.37434400

C -3.77609300 2.60259000 0.27747900

C -2.84833800 -2.31053100 -0.17995400

C -3.42849300 -3.35418100 0.55547000

C -4.80861000 -3.46859900 0.66349900

C -5.63854900 -2.54681600 0.02816800

C -5.07452400 -1.51760500 -0.72211600

C -3.69262400 -1.39803400 -0.82708500

C 4.33895600 0.10666000 -0.88373800

O 5.43227100 -0.39896300 -0.92768900

O 4.03785200 1.28966100 -1.44091900

C 5.13573000 1.96844800 -2.07907000

C 3.41649200 -0.40122700 1.33667800

O 3.42421400 -1.30361600 2.12928100

O 3.62464100 0.88805000 1.66235200

C 3.86744800 1.14573200 3.05853300

H 3.60742800 -2.65909600 0.15072500

H 3.38798200 -2.22601400 -1.54737400

H 1.50977600 0.90608200 0.19801600

H 1.75447800 0.51951000 -1.49246000

H -0.65846000 3.78033200 -0.40727900

H -1.86797800 5.93397200 -0.22842800

H -4.29659200 5.95742000 0.27033200

H -5.51102200 3.81948800 0.59488800

H -4.29779400 1.66465600 0.42276200

H -2.78108100 -4.06871200 1.05094300

H -5.23722100 -4.27700400 1.24438600

H -6.71555400 -2.63548600 0.11008700

H -5.71216000 -0.80859600 -1.23774800

H -3.26798800 -0.60573700 -1.42672300

H 4.71523200 2.88866900 -2.47675100

H 5.91827400 2.18286100 -1.35034200

H 5.55003900 1.35295300 -2.87807300

H 4.03305600 2.21770600 3.13032500

H 3.00312600 0.84737700 3.65287600

H 4.74458500 0.59348600 3.39697800

**Free Energy and Geometry for IN2**

| Zero-point correction= | 0.744148 (Hartree/Particle) |
| --- | --- |
| Thermal correction to Energy= | 0.790485 |
| Thermal correction to Enthalpy= | 0.791429 |
| Thermal correction to Gibbs Free Energy= | 0.659342 |
| Sum of electronic and zero-point Energies= | -2256.108153 |
| Sum of electronic and thermal Energies= | -2256.061816 |
| Sum of electronic and thermal Enthalpies= | -2256.060872 |
| Sum of electronic and thermal Free Energies= | -2256.192959 |

Cartesian Coordinates:

C 0.63967800 -1.60396200 0.49517500

C 1.86495500 -0.92064800 0.39348100

C 1.99336300 0.47923100 0.45923300

C 0.82033200 1.29076100 0.60566300

C -0.35080500 0.57853700 0.77519300

C -0.46491700 -0.80696200 0.73929200

C 0.83389300 -3.08257300 0.27719500

C 2.23860500 -3.14324500 -0.39281000

C 2.98271100 -1.90804800 0.17208600

C 3.28612300 1.05403500 0.38153500

C 4.38949200 1.54592300 0.31731300

C -1.87891000 0.70478000 0.75818300

N -1.91452800 -0.84318800 0.72192200

O -2.50737100 1.11908200 1.95030900

C -2.47390800 0.00960100 2.85368700

C -2.69529100 -1.20144000 1.93619700

C -2.49170400 -2.60228500 2.54734700

C -2.58692400 -3.64896300 1.42606300

C -3.64779100 -2.84756400 3.53727100

C -1.16569400 -2.74836900 3.31479500

C 2.18941500 -3.01443600 -1.92103400

O 3.17372500 -2.83571200 -2.59248400

O 0.95095000 -3.11020000 -2.42717900

C 0.85912800 -2.99366600 -3.85861900

C 2.93072500 -4.45368800 -0.03680900

O 3.69860900 -4.60986800 0.87580200

O 2.51732700 -5.44393600 -0.84910900

C 3.08227300 -6.74281800 -0.59083900

C 5.64489900 2.20094200 0.22717200

C 5.72038300 3.59479300 0.40349200

C 6.94261000 4.24676600 0.30762100

C 8.10553000 3.52678100 0.03660900

C 8.03973400 2.14533400 -0.13893900

C 6.82235900 1.48347000 -0.04527700

C 0.81037100 2.76652600 0.57028800

C 1.60525600 3.49266900 -0.32692000

C 1.53503600 4.88075800 -0.37067300

C 0.67206500 5.56946500 0.47935900

C -0.12962100 4.85722100 1.36800500

C -0.06416600 3.46912800 1.41181500

H 0.07042200 -3.53864800 -0.34739400

H 0.85900400 -3.62183500 1.23028200

H 3.46657700 -2.17812900 1.11517900

H 3.75455500 -1.55409200 -0.50935600

H -3.26262400 0.16431200 3.58841600

H -1.50526600 -0.03812700 3.36217800

H -3.73537000 -1.16028800 1.60284100

H -2.51386300 -4.65818900 1.84086200

H -1.79870800 -3.52353800 0.68625400

H -3.54369000 -3.56629300 0.90291800

H -3.56566400 -3.84811900 3.96977000

H -3.63417800 -2.13050400 4.36224100

H -4.61787000 -2.77519700 3.03781900

H -0.30156900 -2.46389600 2.71824600

H -1.15802600 -2.13388500 4.21819400

H -1.03396900 -3.78757300 3.62825200

H -0.20208800 -3.05506200 -4.08668100

H 1.27297900 -2.03986800 -4.18667800

H 1.40689400 -3.80610000 -4.33749500

H 2.64723500 -7.40159600 -1.33811600

H 4.16749300 -6.70765100 -0.69055600

H 2.82475100 -7.07498800 0.41559000

H 4.81260500 4.14625900 0.61559300

H 6.98940900 5.32084800 0.44570500

H 9.05754500 4.03923500 -0.03685900

H 8.94123400 1.58182900 -0.34980900

H 6.76616000 0.41074400 -0.18244000

H 2.26577700 2.96790000 -1.00293400

H 2.15064000 5.42474100 -1.07798600

H 0.62101000 6.65160800 0.44480800

H -0.80791600 5.38294100 2.03031000

H -0.69561400 2.91970500 2.09924500

C -2.45136700 1.40074900 -0.42262000

C -3.05846200 2.69152300 -0.43108200

C -2.40083500 0.92552100 -1.76669900

C -3.39544500 3.00533500 -1.77739900

H -3.25286300 3.29257000 0.44132300

C -2.98915400 1.91566800 -2.60181200

H -2.02197200 -0.03770900 -2.06838900

Fe -4.35028900 1.26335600 -1.18072400

H -3.90468300 3.89650800 -2.10980500

H -3.14160100 1.83617900 -3.66701900

C -6.27267700 1.54317000 -0.46240900

C -6.31492000 1.19667000 -1.84397400

C -5.62890600 0.48279400 0.23861600

H -6.62958100 2.46385300 -0.02772700

C -5.69728400 -0.07853000 -1.99650300

H -6.71167500 1.80840500 -2.63929300

C -5.27183000 -0.52064800 -0.70998900

H -5.40099100 0.47592400 1.29248900

H -5.54178700 -0.59999100 -2.92815000

H -4.71939500 -1.42398000 -0.50485600

**Free Energy and Geometry for TS1**

| Zero-point correction= | 0.742119 (Hartree/Particle) |
| --- | --- |
| Thermal correction to Energy= | 0.788068 |
| Thermal correction to Enthalpy= | 0.789012 |
| Thermal correction to Gibbs Free Energy= | 0.659399 |
| Sum of electronic and zero-point Energies= | -2256.087302 |
| Sum of electronic and thermal Energies= | -2256.041353 |
| Sum of electronic and thermal Enthalpies= | -2256.040409 |
| Sum of electronic and thermal Free Energies= | -2256.170021 |

Cartesian Coordinates:

C 0.19896400 1.45755400 0.87628200

C -1.13082900 1.17816400 0.58634800

C -1.66653300 -0.12278500 0.62399300

C -0.76847600 -1.21275800 0.82877900

C 0.57299900 -0.89446000 1.04249300

C 1.08339600 0.41124300 1.16261900

C 0.49280200 2.92630000 0.74719800

C -0.69994200 3.42709500 -0.11625100

C -1.86116600 2.44223200 0.21025200

C -3.05481800 -0.31995600 0.44251300

C -4.24372800 -0.49582800 0.29405900

C 1.75669300 -1.79225900 0.87052900

N 2.45055200 0.50527900 1.31131500

O 2.50084900 -2.18559500 1.88106800

C 2.51005900 -1.29618800 3.01465700

C 3.07894600 0.06215100 2.52008100

C 3.10289800 1.11191900 3.69279200

C 3.78245800 2.38231200 3.16092700

C 3.93042200 0.56455800 4.86941900

C 1.69040100 1.46458700 4.18165700

C -0.29327600 3.27909400 -1.58220500

O -0.64843800 2.40128500 -2.32708800

O 0.59107100 4.23472800 -1.92024800

C 1.12522800 4.15418400 -3.25466400

C -1.08968600 4.86359600 0.18824600

O -0.70834800 5.51644400 1.12425000

O -1.98409300 5.30458300 -0.71872500

C -2.45340800 6.65056700 -0.52782800

C -5.61548500 -0.80283800 0.10782100

C -6.05894400 -2.13129400 0.25135700

C -7.39742500 -2.44832300 0.06238200

C -8.31761600 -1.45486700 -0.27056000

C -7.88806900 -0.13634200 -0.41390100

C -6.55130000 0.19150400 -0.22765200

C -1.20414500 -2.62610500 0.77762200

C -2.07235800 -3.09734700 -0.21779800

C -2.42626000 -4.43981500 -0.27369300

C -1.92332800 -5.34102900 0.66407600

C -1.06326700 -4.88647100 1.65912000

C -0.70871300 -3.54207600 1.71469100

H 1.45950800 3.13537900 0.28926700

H 0.47650500 3.44265800 1.71176600

H -2.45416800 2.81753400 1.05162300

H -2.52858700 2.30644200 -0.63930300

H 3.13297100 -1.78926500 3.75501700

H 1.49040000 -1.18968300 3.38739600

H 4.13495800 -0.11735900 2.27408800

H 3.82613500 3.14794100 3.94121000

H 3.23660200 2.77719700 2.30471300

H 4.80546300 2.16917200 2.83569700

H 4.09660700 1.35019200 5.61186900

H 3.43089600 -0.26148200 5.38320100

H 4.91153600 0.21434500 4.53296000

H 1.08162600 1.87371900 3.37627600

H 1.16448100 0.59939100 4.59403400

H 1.74871000 2.21649600 4.97401100

H 1.73893900 5.04359000 -3.37414000

H 1.73147600 3.25376000 -3.36288700

H 0.31686000 4.13806100 -3.98605100

H -3.15531200 6.83133900 -1.33822100

H -2.94699400 6.74771900 0.43998500

H -1.61897400 7.35144000 -0.57533200

H -5.33961800 -2.89769100 0.51306800

H -7.72589600 -3.47509700 0.17673300

H -9.36162800 -1.70618300 -0.41607400

H -8.59895100 0.64015200 -0.67230000

H -6.21441300 1.21462900 -0.34010900

H -2.45901200 -2.40887700 -0.95691600

H -3.09165900 -4.78514700 -1.05670700

H -2.20103500 -6.38762200 0.61849000

H -0.67117300 -5.57640000 2.39736800

H -0.05518700 -3.19332100 2.50471000

C 2.00964700 -2.45010500 -0.37339100

C 3.21484100 -3.15195900 -0.73805400

C 1.11834900 -2.50228400 -1.51032500

C 3.04215000 -3.65882300 -2.04668300

H 4.08589800 -3.25436900 -0.11239100

C 1.74927500 -3.27000400 -2.51582300

H 0.14637900 -2.04685300 -1.56582300

Fe 2.85634200 -1.57859300 -2.04958800

H 3.77710800 -4.21171100 -2.61076600

H 1.34115700 -3.48346200 -3.49116000

C 4.51252500 -0.33438400 -2.14808000

C 4.18184100 -0.82786900 -3.44619000

C 3.39323500 0.38737100 -1.64878900

H 5.43627500 -0.51159200 -1.61947800

C 2.85424900 -0.40660100 -3.74711800

H 4.81100500 -1.43777400 -4.07611300

C 2.36312400 0.34048900 -2.63425600

H 3.28784200 0.77911600 -0.64369200

H 2.30006200 -0.65230200 -4.63998700

H 1.37173800 0.75793200 -2.53989700

**Free Energy and Geometry for IN3**

| Zero-point correction= | 0.743335 (Hartree/Particle) |
| --- | --- |
| Thermal correction to Energy= | 0.790037 |
| Thermal correction to Enthalpy= | 0.790981 |
| Thermal correction to Gibbs Free Energy= | 0.659395 |
| Sum of electronic and zero-point Energies= | -2256.119348 |
| Sum of electronic and thermal Energies= | -2256.072646 |
| Sum of electronic and thermal Enthalpies= | -2256.071702 |
| Sum of electronic and thermal Free Energies= | -2256.203288 |

Cartesian Coordinates:

C 0.07648700 -1.55926900 1.10158900

C -1.12758500 -1.12515200 0.65927600

C -1.49660000 0.27724100 0.65564300

C -0.48941200 1.21777500 0.79970800

C 0.87465600 0.73001000 0.98467500

C 1.07891400 -0.62206100 1.55927500

C 0.15896400 -3.06006700 1.12136300

C -0.99025900 -3.44555400 0.14833700

C -2.01578400 -2.28350100 0.28362300

C -2.86874000 0.59888600 0.55213600

C -4.05301800 0.83505600 0.46180200

C 1.95650800 1.28379100 0.33114300

O 3.23792300 0.90558300 0.52660400

C 3.85018100 0.56617400 1.79491700

C 2.87708800 0.07583400 2.85284200

N 2.05689100 -0.99492800 2.30903900

C 3.58166900 -0.39375500 4.15898500

C 4.46375700 -1.62454200 3.89948400

C 2.47814400 -0.76941300 5.16145900

C 4.42471300 0.74714300 4.75194900

C -1.62056400 -4.78864300 0.48168000

O -1.49845800 -5.38513000 1.51907500

O -2.39307700 -5.20960400 -0.53948200

C -3.07145100 -6.46151900 -0.33414300

C -0.44549600 -3.45870300 -1.28405800

O -0.75456100 -2.69554800 -2.16390300

O 0.47106400 -4.43071800 -1.42914400

C 1.08585700 -4.52335500 -2.72884200

C -5.41500200 1.21009100 0.34082000

C -5.79303100 2.55069100 0.54331600

C -7.12236900 2.93028700 0.41516600

C -8.09593000 1.98783900 0.08590400

C -7.73106300 0.65722600 -0.11462000

C -6.40432100 0.26699000 0.01109200

C -0.74243600 2.67278700 0.86309400

C -0.00679700 3.46347600 1.75918100

C -0.22328900 4.83336200 1.85255300

C -1.17616100 5.44853300 1.04536000

C -1.91076100 4.67792400 0.14558000

C -1.69907900 3.30802300 0.05628700

H -0.04526900 -3.47018300 2.11420500

H 1.12599100 -3.45020600 0.80521600

H -2.56231500 -2.11969200 -0.64390100

H -2.74155400 -2.49297100 1.07715300

H 4.56400400 -0.21497000 1.53936600

H 4.39545600 1.45474500 2.12516200

H 2.23819200 0.93042200 3.12668800

H 4.85304100 -2.01421800 4.84407100

H 5.32399700 -1.38817400 3.26659200

H 3.88192300 -2.40758800 3.41183800

H 2.91738700 -1.11258900 6.10224000

H 1.83913700 0.09207200 5.38041900

H 1.84897200 -1.56390300 4.75824600

H 3.82990700 1.65829400 4.87285300

H 4.79817200 0.46266400 5.73933200

H 5.29374300 0.98772000 4.13404700

H -3.63120500 -6.64176600 -1.24856100

H -2.34786200 -7.25867500 -0.15938000

H -3.74258600 -6.39381300 0.52301300

H 1.74117900 -5.38922600 -2.67652300

H 0.32417000 -4.65830000 -3.49730500

H 1.65769200 -3.61920300 -2.94103900

H -5.03187200 3.27600600 0.80341500

H -7.40184800 3.96540300 0.57443300

H -9.13246800 2.28818600 -0.01212700

H -8.48455500 -0.07891800 -0.36984200

H -6.11620100 -0.76514900 -0.14543600

H 0.72992000 2.99083400 2.39552200

H 0.35186900 5.42034300 2.55942800

H -1.34274000 6.51730000 1.11278800

H -2.64453600 5.14768100 -0.49951700

H -2.26280800 2.72832000 -0.66047800

C 1.85641800 2.13152400 -0.85185400

C 0.76182200 2.20317100 -1.78336000

C 2.95939600 2.84448900 -1.43541900

C 1.18082100 2.97577400 -2.89526500

H -0.18812600 1.71162100 -1.67777200

C 2.53637600 3.36704800 -2.68383100

H 3.94016700 2.93131400 -0.99877300

Fe 2.36679800 1.28830900 -2.65522100

H 0.58815100 3.19006200 -3.77070500

H 3.14593400 3.93353200 -3.37047200

C 1.72414700 -0.59490200 -3.23080000

C 2.21718500 0.12417900 -4.36008400

C 2.78651400 -0.69901100 -2.28616200

H 0.72196100 -0.97037800 -3.09279600

C 3.58036300 0.46138100 -4.11277100

H 1.64629700 0.39691600 -5.23409400

C 3.93230800 -0.04628300 -2.82703700

H 2.71791900 -1.13466600 -1.30119700

H 4.22160200 1.03103400 -4.76739600

H 4.88223600 0.07607800 -2.33140400

**Free Energy and Geometry for TS2**

| Zero-point correction= | 0.762354 (Hartree/Particle) |
| --- | --- |
| Thermal correction to Energy= | 0.810902 |
| Thermal correction to Enthalpy= | 0.811846 |
| Thermal correction to Gibbs Free Energy= | 0.676658 |
| Sum of electronic and zero-point Energies= | -2332.474178 |
| Sum of electronic and thermal Energies= | -2332.425630 |
| Sum of electronic and thermal Enthalpies= | -2332.424686 |
| Sum of electronic and thermal Free Energies= | -2332.559875 |

Cartesian Coordinates:

C 0.48754800 0.97805800 -1.13653400

C -0.83273500 0.89414900 -0.85856200

C -1.54873900 -0.37511900 -0.74081700

C -0.85566600 -1.55327700 -0.76729200

C 0.63678000 -1.54514600 -0.97912900

C 1.30532000 -0.18911200 -1.33787600

C 0.92214200 2.39552200 -1.38264500

C -0.27257000 3.22913600 -0.83641200

C -1.48303700 2.25009200 -0.81499300

C -2.95962400 -0.31738400 -0.61665300

C -4.16145400 -0.24080000 -0.50105500

C 1.43681100 -1.91782600 0.20592000

O 2.69223200 -2.23926000 0.07251600

C 3.27631300 -2.47705000 -1.24928800

C 3.24638000 -1.25259900 -2.19752400

N 2.49439300 -0.12120700 -1.82154800

C 4.63771800 -0.85804400 -2.74153400

C 5.50980900 -0.26012100 -1.62305900

C 4.44653800 0.19022200 -3.85015100

C 5.32811100 -2.08830400 -3.35911000

C -0.57716100 4.44041400 -1.70903200

O -0.14498400 4.64378800 -2.81220700

O -1.45955700 5.24777500 -1.08723200

C -1.86136500 6.41712100 -1.82328200

C 0.04475500 3.69714800 0.58364400

O -0.47852000 3.30125400 1.59505700

O 1.03544200 4.60474900 0.56005900

C 1.45200100 5.12880500 1.83463900

C -5.57046400 -0.25908400 -0.33645300

C -6.25897200 -1.48591600 -0.36496600

C -7.63606100 -1.51873000 -0.19050900

C -8.34830700 -0.33737300 0.01279800

C -7.67418300 0.88268700 0.04047400

C -6.29691200 0.92665600 -0.13270700

C -1.45457500 -2.89060500 -0.62972400

C -0.97374300 -3.95981100 -1.40887000

C -1.52979500 -5.22837100 -1.27929600

C -2.54941100 -5.46514200 -0.36093700

C -3.01623800 -4.41803700 0.43276400

C -2.47788900 -3.14479200 0.29911800

H 1.04568800 2.58543800 -2.45191100

H 1.87066300 2.65890400 -0.91332400

H -2.09619300 2.40680600 0.07249500

H -2.12641000 2.39590300 -1.68963400

H 4.28906100 -2.77439200 -0.99400500

H 2.74567000 -3.31886200 -1.69329500

H 0.86954300 -2.17902300 -1.87805700

H 6.48971900 0.04479700 -2.00369000

H 5.68164800 -0.98178800 -0.81721600

H 5.01794300 0.61743700 -1.19603300

H 5.41560700 0.49613600 -4.25575200

H 3.84802900 -0.22252600 -4.66712100

H 3.93151300 1.07039500 -3.46576500

H 4.66781800 -2.57540000 -4.08140100

H 6.24392300 -1.78743500 -3.87543500

H 5.61389200 -2.83231700 -2.60975700

H -2.56295900 6.93959300 -1.17766900

H -0.99490000 7.04295900 -2.04036400

H -2.33888900 6.12974100 -2.76079300

H 2.15961400 5.92091000 1.60304600

H 0.59335100 5.52166700 2.37975400

H 1.93294600 4.34721900 2.42342600

H -5.69818100 -2.39819200 -0.52733900

H -8.15706000 -2.46883000 -0.21522600

H -9.42316600 -0.36722100 0.14718200

H -8.22482800 1.80294100 0.19725500

H -5.76823100 1.87157700 -0.11044000

H -0.19216000 -3.79163100 -2.14420900

H -1.16083600 -6.03531800 -1.90194400

H -2.97198000 -6.45799500 -0.25739200

H -3.79534500 -4.59560000 1.16551600

H -2.83407900 -2.34423800 0.93179800

C 1.00700400 -1.86517600 1.57394400

C -0.09236900 -1.14175700 2.16395200

C 1.73592700 -2.49611800 2.65465900

C -0.04988400 -1.35668300 3.55990900

H -0.78749700 -0.51033700 1.64336100

C 1.06352600 -2.19773100 3.86054100

H 2.61983100 -3.09910100 2.53238900

Fe 1.71933800 -0.46530800 2.89713700

H -0.72220700 -0.91686600 4.27971200

H 1.36638000 -2.51725200 4.84541000

C 1.70342200 1.57963700 3.22025700

C 2.43932100 0.92550400 4.25087600

C 2.32937800 1.27654100 1.97869100

H 0.79775500 2.15357500 3.33323400

C 3.52460800 0.22578400 3.64328300

H 2.19763800 0.92857300 5.30243300

C 3.45537400 0.43962100 2.23866300

H 1.98307800 1.57282100 1.00367200

H 4.24066700 -0.39870500 4.15472100

H 4.10123500 0.00484200 1.49141200

O 1.51975000 -2.68681600 -3.45051700

H 1.09827500 -2.36986900 -4.25670900

H 2.50920500 -1.81674000 -3.07278100

**Free Energy and Geometry for TS3**

| Zero-point correction= | 0.737572 (Hartree/Particle) |
| --- | --- |
| Thermal correction to Energy= | 0.783860 |
| Thermal correction to Enthalpy= | 0.784804 |
| Thermal correction to Gibbs Free Energy= | 0.654459 |
| Sum of electronic and zero-point Energies= | -2256.047365 |
| Sum of electronic and thermal Energies= | -2256.001077 |
| Sum of electronic and thermal Enthalpies= | -2256.000133 |
| Sum of electronic and thermal Free Energies= | -2256.130478 |

Cartesian Coordinates:

C -0.32832700 1.19297100 1.19791600

C 0.90836800 0.77490700 0.80180500

C 1.25211000 -0.61779900 0.62509200

C 0.26159100 -1.58781400 0.64418300

C -1.13443700 -1.17336600 0.95492600

C -1.33630800 0.23905100 1.49461100

C -0.35032000 2.66126100 1.51633400

C 0.96468100 3.17534500 0.86684300

C 1.88419000 1.92290700 0.76431400

C 2.62813000 -0.91444200 0.46114900

C 3.81474600 -1.11489600 0.32998100

C -2.10151200 -1.33051700 -0.10840800

O -3.38861400 -1.08667800 0.16211000

C -3.88333000 -1.38114900 1.49995700

C -3.02042900 -0.78493700 2.58887700

N -2.32677000 0.37682800 2.32973500

C -3.55957900 -0.92198100 4.01556600

C -4.67809500 0.11923500 4.24465800

C -2.41242600 -0.65420000 5.00469800

C -4.11053500 -2.33707200 4.26414800

C 1.62934000 4.26163600 1.70407200

O 1.40235100 4.49423600 2.86202500

O 2.56378800 4.90777500 0.97959200

C 3.29612800 5.93178400 1.67607700

C 0.69037100 3.71996800 -0.53715000

O 1.17065300 3.30649700 -1.56216500

O -0.18580400 4.73710700 -0.48297200

C -0.53288100 5.33762600 -1.74570800

C 5.18321100 -1.44322800 0.15236200

C 5.59419400 -2.78810400 0.20284300

C 6.93029600 -3.11945400 0.02100800

C 7.87765700 -2.12335400 -0.21221000

C 7.47982200 -0.78809300 -0.26308600

C 6.14596300 -0.44611300 -0.08292300

C 0.46701600 -3.01731000 0.38290300

C -0.39870800 -3.96381400 0.96219700

C -0.25496900 -5.32412400 0.70983100

C 0.74599700 -5.77833900 -0.14219500

C 1.60240700 -4.85377400 -0.74124200

C 1.46726800 -3.49734300 -0.48461300

H -0.31670600 2.82794500 2.59637100

H -1.22955600 3.18490000 1.14107900

H 2.48733500 1.95510300 -0.14223500

H 2.56771900 1.86684000 1.61866500

H -4.88814100 -0.94876100 1.47828400

H -3.98429800 -2.46747300 1.60591600

H -1.72984100 -1.66341400 1.97179600

H -5.04515300 0.06529300 5.27411000

H -5.52871600 -0.05246600 3.57837500

H -4.29526400 1.12427400 4.06066100

H -2.77688000 -0.70501100 6.03493000

H -1.61860400 -1.39626000 4.88147900

H -1.98114800 0.33217100 4.83224200

H -3.35740500 -3.09660100 4.03391100

H -4.38568200 -2.44715900 5.31619600

H -5.00487100 -2.54721800 3.67139900

H 3.99038600 6.33962700 0.94557700

H 2.61572500 6.70383800 2.03731600

H 3.83335300 5.50383600 2.52333800

H -1.16386700 6.18720200 -1.49660600

H 0.36722700 5.66124300 -2.26892100

H -1.07706100 4.62261400 -2.36332900

H 4.85353800 -3.55600600 0.38951400

H 7.23534400 -4.15869400 0.06351600

H 8.91960900 -2.38594000 -0.35227700

H 8.21287600 -0.01046500 -0.44398700

H 5.83193100 0.58954200 -0.12358600

H -1.17338700 -3.63367200 1.64122600

H -0.92906100 -6.02958300 1.18225900

H 0.85495900 -6.83727700 -0.34527600

H 2.37292800 -5.19157600 -1.42507400

H 2.12366500 -2.79879400 -0.98104500

C -1.84983100 -1.56829900 -1.50042500

C -0.63273300 -1.38483300 -2.25096900

C -2.88431500 -1.91809300 -2.44553000

C -0.91456100 -1.65817100 -3.61171800

H 0.31130500 -1.06030400 -1.85336900

C -2.29726900 -1.98984800 -3.73143000

H -3.91708600 -2.08793100 -2.19236700

Fe -2.08826200 -0.08571400 -2.91675600

H -0.20940100 -1.58197400 -4.42446100

H -2.81589200 -2.21715700 -4.64972500

C -1.28102200 1.81844200 -3.00948000

C -1.95577200 1.51211500 -4.22769600

C -2.23151000 1.73163600 -1.95158700

H -0.23413600 2.04674900 -2.89406000

C -3.32092200 1.23748000 -3.92208900

H -1.50314700 1.46092500 -5.20579000

C -3.49213300 1.37022900 -2.51294500

H -2.02009300 1.84560800 -0.89998400

H -4.08041000 0.93726200 -4.62732000

H -4.39822500 1.18128000 -1.95966500

**Free Energy and Geometry for 3a**

| Zero-point correction= | 0.743637 (Hartree/Particle) |
| --- | --- |
| Thermal correction to Energy= | 0.790311 |
| Thermal correction to Enthalpy= | 0.791255 |
| Thermal correction to Gibbs Free Energy= | 0.658435 |
| Sum of electronic and zero-point Energies= | -2256.139954 |
| Sum of electronic and thermal Energies= | -2256.093281 |
| Sum of electronic and thermal Enthalpies= | -2256.092337 |
| Sum of electronic and thermal Free Energies= | -2256.225156 |

Cartesian Coordinates:

C -1.66591800 -1.54332800 0.61025100

C -2.35290500 -0.33870600 0.60807400

C -1.68847700 0.86313600 0.34209800

C -0.28729500 0.84014700 0.10017900

C 0.41103500 -0.37273800 0.13477800

C -0.29963600 -1.58405100 0.34676000

C -2.59846800 -2.69412700 0.88440800

C -3.99462500 -2.07207400 0.58053200

C -3.81690500 -0.55933800 0.88363200

C -2.39328200 2.09028000 0.26821800

C -2.93040800 3.16897200 0.16906600

C 1.92371500 -0.54038500 0.00199400

O 2.28810100 -1.05170900 -1.29323500

C 1.44728500 -2.11546800 -1.73674100

C 1.14871900 -3.12061600 -0.62750100

N 0.32441500 -2.84261600 0.31155000

C 1.83997100 -4.47728000 -0.64626100

C 1.37877500 -5.24800500 -1.90292700

C 3.36661100 -4.26320700 -0.69894500

C 1.48223200 -5.29053100 0.60306000

C -5.06458800 -2.71656400 1.45319500

O -5.49714900 -2.26320600 2.47979300

O -5.42634700 -3.91106200 0.94804100

C -6.41659800 -4.63434900 1.70181300

C -4.41806200 -2.21587000 -0.88610600

O -5.40008400 -1.68287700 -1.33731200

O -3.58238800 -2.97202700 -1.61609500

C -3.94183700 -3.12165200 -3.00118700

C -3.45166200 4.48009400 0.02099700

C -2.59225000 5.51697600 -0.38828400

C -3.08073100 6.80764500 -0.53949100

C -4.42387200 7.08725300 -0.28865300

C -5.28100900 6.06508800 0.11608000

C -4.80338200 4.76984900 0.27098800

C 0.33800900 2.14021800 -0.27985800

C 0.52125600 2.44135200 -1.63077400

C 1.01534100 3.68347400 -2.01740700

C 1.33644400 4.63599200 -1.05355000

C 1.15364800 4.34144800 0.29620900

C 0.64728800 3.10499100 0.67960700

H -2.40104200 -3.57947300 0.28802400

H -2.53850400 -2.97938200 1.94086700

H -4.48036700 0.05749800 0.27850100

H -4.05605300 -0.37108200 1.93415000

H 2.18816600 -1.30475600 0.74320900

H 1.97154000 -2.57226800 -2.57322100

H 0.49188000 -1.71707700 -2.10218500

H 1.86009000 -6.22901700 -1.93143200

H 0.29657400 -5.40048100 -1.88755500

H 1.63911800 -4.72584000 -2.82662300

H 3.87443300 -5.23102600 -0.71281000

H 3.71376400 -3.71146500 0.17716900

H 3.67516200 -3.71169000 -1.58880200

H 1.78559900 -4.76753300 1.51141300

H 0.40694100 -5.46018800 0.66840800

H 1.99084600 -6.25787200 0.56948900

H -6.58433200 -5.55619500 1.15055300

H -6.04881900 -4.84546500 2.70663500

H -7.33607000 -4.05223700 1.77091800

H -3.15754200 -3.73376100 -3.43998500

H -4.91138300 -3.61332000 -3.08792600

H -3.99107800 -2.14622000 -3.48616000

H -1.55125600 5.28873300 -0.58106300

H -2.41158000 7.59984800 -0.85528100

H -4.80076500 8.09627300 -0.40848700

H -6.32561700 6.27838600 0.31112100

H -5.46504900 3.97199000 0.58468600

H 0.27125000 1.69545300 -2.37610600

H 1.15068500 3.90640400 -3.06961400

H 1.72722800 5.60221200 -1.35161900

H 1.40371800 5.07720500 1.05160200

H 0.49715000 2.87823900 1.72757300

C 2.78356800 0.64219400 0.31020700

C 3.41590800 1.54002700 -0.59437000

C 3.12911500 1.04356400 1.63696000

C 4.13981100 2.49792400 0.16753500

H 3.36407900 1.47785400 -1.66743200

C 3.96352900 2.19303400 1.54759900

H 2.82214500 0.54480200 2.54349100

Fe 4.82653800 0.58398100 0.56700000

H 4.74432500 3.29616400 -0.23354000

H 4.41244700 2.71672200 2.37703000

C 6.37021600 -0.05548500 -0.65092100

C 6.90059700 0.52226800 0.53910600

C 5.54337000 -1.15418800 -0.27884200

H 6.52946600 0.30398500 -1.65559900

C 6.40211600 -0.22010000 1.64869700

H 7.53944600 1.39013900 0.59293500

C 5.56439300 -1.25773100 1.14288900

H 4.94413600 -1.74704500 -0.94902800

H 6.60009200 -0.01499900 2.68936700

H 5.01497700 -1.97246800 1.73626400

1. **Photophysical data**

**Table S1**. Spectroscopic and photophysical properties of compounds **3j** and **4c** in different solvents.

| Dyes | Solvent | λ_abs_^max^ (nm) | ε^a^ | λ_em_^max^ (nm) | Φ^b^ | Stokes shift  (cm^-1^) |
| --- | --- | --- | --- | --- | --- | --- |
| **3j** | MeCN | 330 | 13400 | 353 | 0.003 | 1970 |
|  | EtOAc | 330 | 11700 | 352 | 0.004 | 1890 |
|  | Toluene | 336 | 9000 | 354 | 0.006 | 1510 |
| **4c** | MeCN | 342 | 18300 | 413 | 0.003 | 5030 |
|  | EtOAc | 342 | 18900 | 412 | 0.006 | 4970 |
|  | Toluene | 346 | 12400 | 407 | 0.003 | 4330 |
| ^a^Data corresponding to the strongest absorption maximum, the unit for ε is M^-1^ cm^-1^. ^b^Quinine sulfate (Φ = 0.54 in 0.05 M H_2_SO_4_) was used as a standard. | | | | | | |

**Absorption and emission spectra of compounds 3j and 4c at different solvents**

**
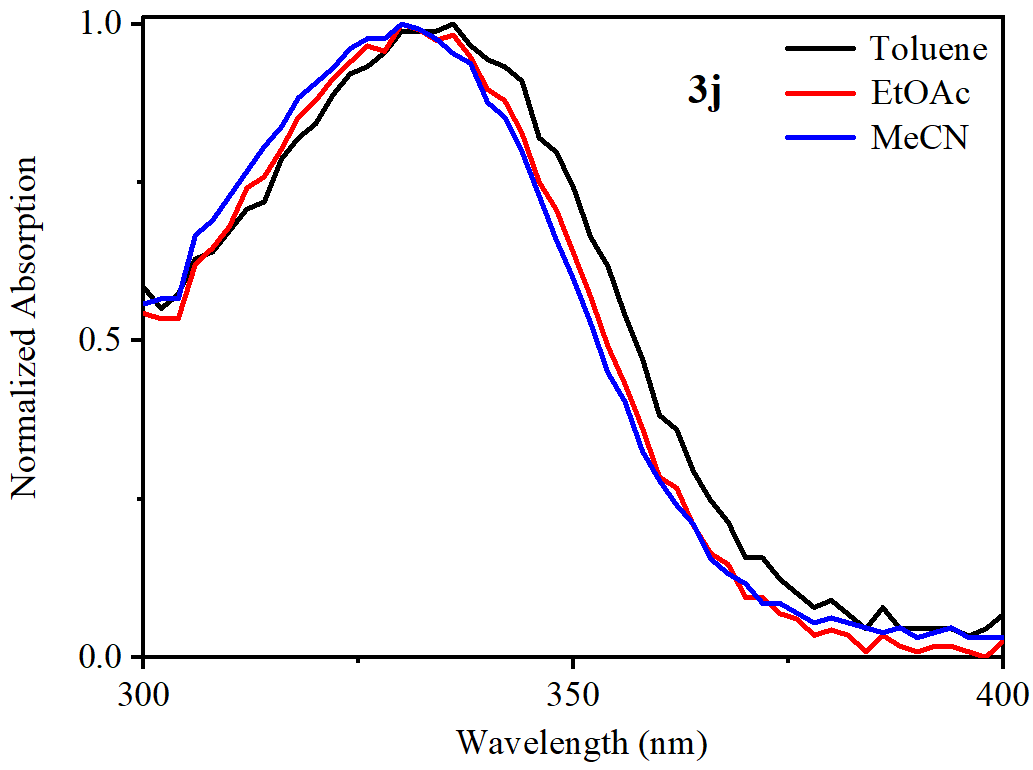
**
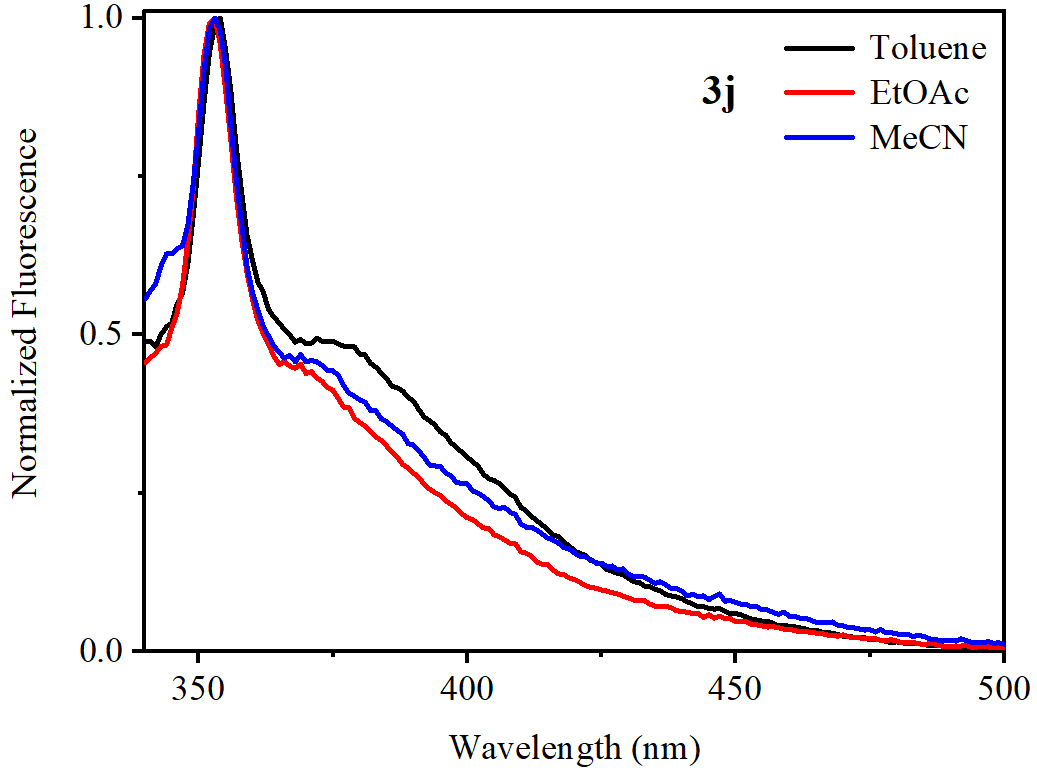


**Figure S4**. Absorption (left) and emission (right) spectra of compound **3j**.


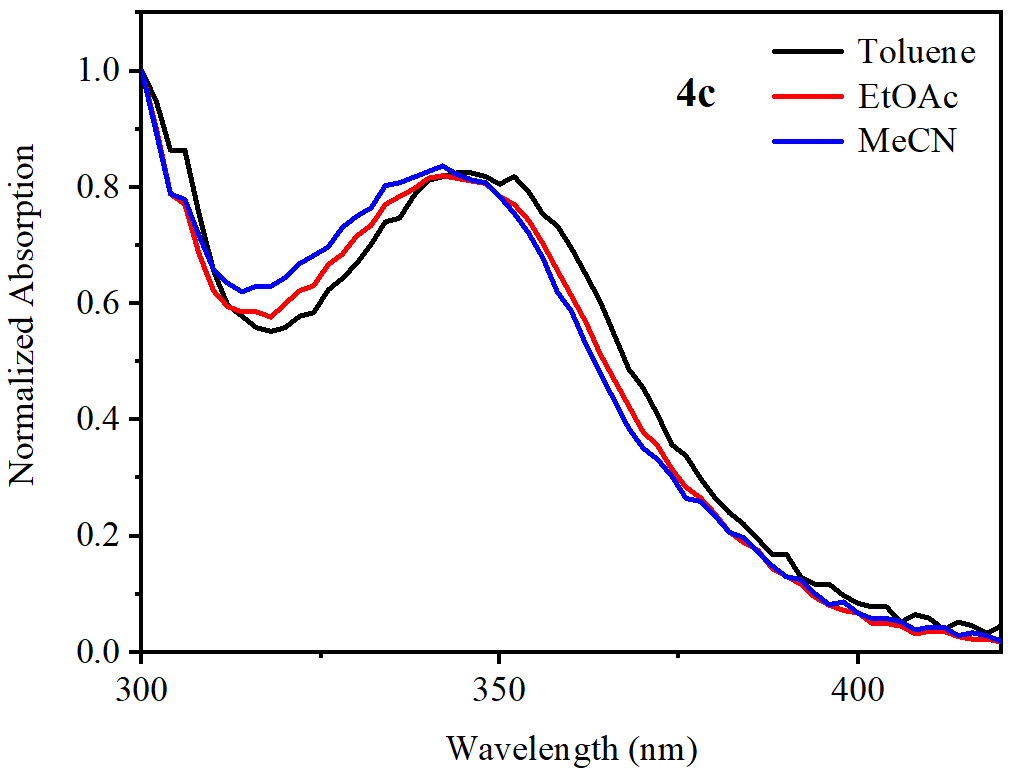

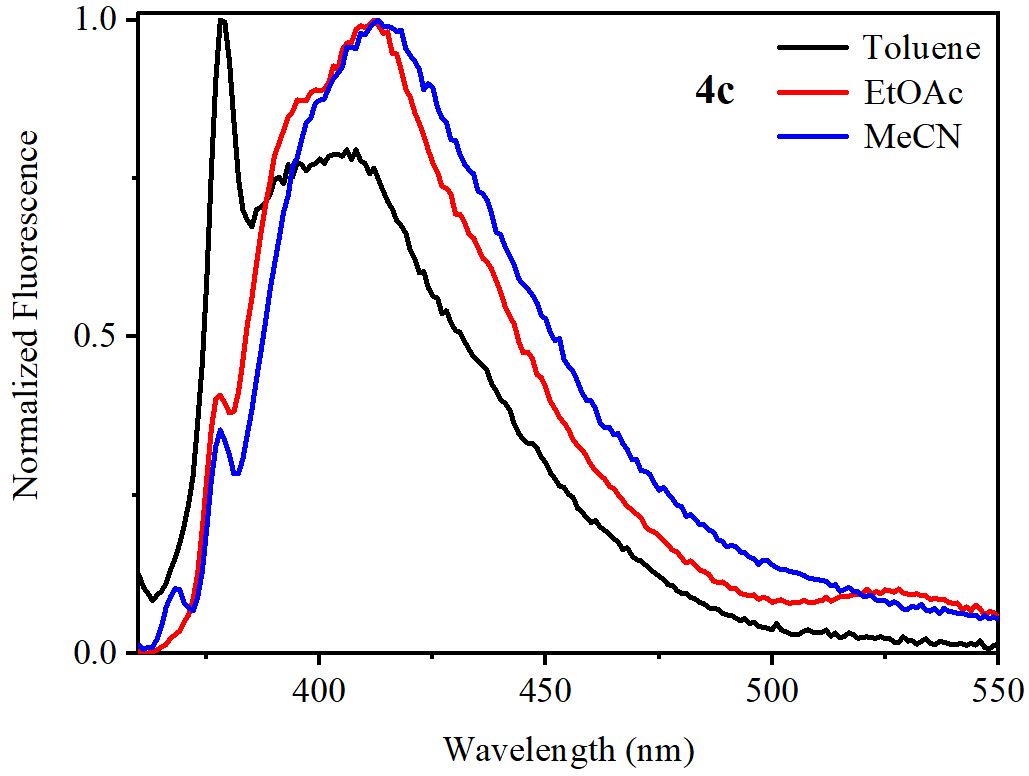


**Figure S5**. Absorption (left) and emission (right) spectra of compound **4c**.

1. **Electrochemical data**

**Table S2**. Electrochemical data acquired at 100 mV/s for compounds **2a**, **3j**, and **4c**.^a^

| Dyes | *E*_1/2_^red^ (V) | *E*_red_^onest^ (V) | *E*_pa_(V) | *E*_ox_^onest^ (V) |
| --- | --- | --- | --- | --- |
| **2a** | -0.90; -0.024 | -0.78 | 0.60 | 0.461 |
| **3j** | -0.88; -0.098 | -0.79 | 0.44 | 0.287 |
| **4c** | -0.85; -0.29 | -0.71 | - | 1.445 |
| ^a^E_1/2_^red^ = half wave potentials of reversible reduction potential; E_red_^onset^ = the onset reduction potentials; E_1/2_^ox^ = half wave potentials of reversible oxidation potential; E_ox_^onset^ = the onset oxidation potentials. | | | | |

**7. ^1^H and ^13^C Spectra for New Compounds**

 ****

 ****

 ****

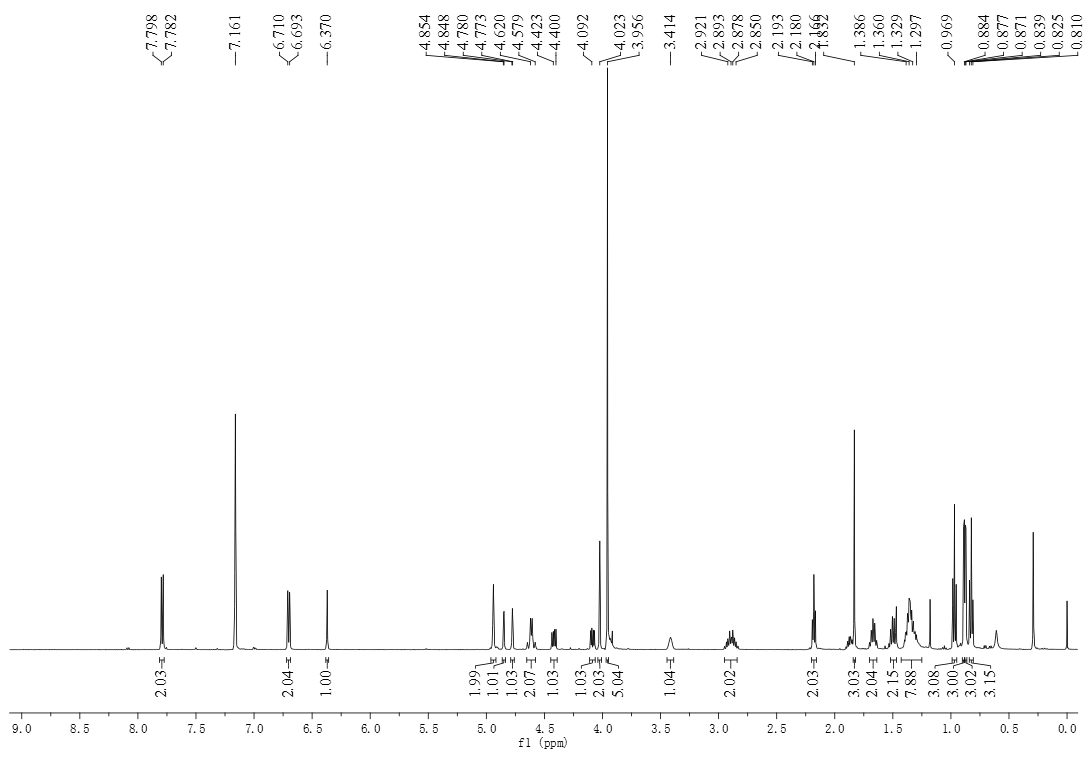

 ****

 ****

 ****

 ****

 ****

 ****

 ****

 ****

 ****

 ****

 ****

 ****

 ****

 ****

 ****

 ****

 ****

 ****

 ****

 ****

 ****

 ****
